# Evaluation of Adjunctive Ultrasonography for Breast Cancer Detection Among Women Aged 40-49 Years With Varying Breast Density Undergoing Screening Mammography: A Secondary Analysis of a Randomized Clinical Trial

**DOI:** 10.1001/jamanetworkopen.2021.21505

**Published:** 2021-08-18

**Authors:** Narumi Harada-Shoji, Akihiko Suzuki, Takanori Ishida, Ying-Fang Zheng, Yoko Narikawa-Shiono, Akiko Sato-Tadano, Rie Ohta, Noriaki Ohuchi

**Affiliations:** 1 Department of Breast and Endocrine Surgical Oncology, Tohoku University Graduate School of Medicine, Sendai, Japan; 2 Department of Breast and Endocrine Surgery, Tohoku Medical and Pharmaceutical University, Sendai, Japan

## Abstract

**Importance:**

Mammography has limited accuracy in breast cancer screening. Ultrasonography, when used in conjunction with mammography screening, is helpful to detect early-stage and invasive cancers for asymptomatic women with dense and nondense breasts.

**Objective:**

To evaluate the performance of adjunctive ultrasonography with mammography for breast cancer screening, according to differences in breast density.

**Design, Setting, and Participants:**

This study is a secondary analysis of the Japan Strategic Anti-cancer Randomized Trial. Between July 2007 and March 2011, asymptomatic women aged 40 to 49 years were enrolled in Japan. The present study used data from cases enrolled from the screening center in Miyagi prefecture during 2007 to 2020. Participants were randomly assigned in a 1:1 ratio to undergo either mammography with ultrasonography (intervention group) or mammography alone (control group). Data analysis was performed from February to March 2020.

**Exposures:**

Ultrasonography adjunctive to mammography for breast cancer screening regardless of breast density.

**Main Outcomes and Measures:**

Sensitivity, specificity, recall rates, biopsy rates, and characteristics of screen-detected cancers and interval breast cancers were evaluated between study groups and for each modality according to breast density.

**Results:**

A total of 76 119 women were enrolled, and data for 19 213 women (mean [SD] age, 44.5 [2.8] years) from the Miyagi prefecture were analyzed; 9705 were randomized to the intervention group and 9508 were randomized to the control group. A total of 11 390 women (59.3%) had heterogeneously or extremely dense breasts. Among the overall group, 130 cancers were found. Sensitivity was significantly higher in the intervention group than the control group (93.2% [95% CI, 87.4%-99.0%] vs 66.7% [95% CI, 54.4%-78.9%]; *P* < .001). Similar trends were observed in women with dense breasts (sensitivity in intervention vs control groups, 93.2% [95% CI, 85.7%-100.0%] vs 70.6% [95% CI, 55.3%-85.9%]; *P* < .001) and nondense breasts (sensitivity in intervention vs control groups, 93.1% [95% CI, 83.9%-102.3%] vs 60.9% [95% CI, 40.9%-80.8%]; *P* < .001). The rate of interval cancers per 1000 screenings was lower in the intervention group compared with the control group (0.5 cancers [95% CI, 0.1-1.0 cancers] vs 2.0 cancers [95% CI, 1.1-2.9 cancers]; *P* = .004). Within the intervention group, the rate of invasive cancers detected by ultrasonography alone was significantly higher than that for mammography alone in both dense (82.4% [95% CI, 56.6%-96.2%] vs 41.7% [95% CI, 15.2%-72.3%]; *P = *.02) and nondense (85.7% [95% CI, 42.1%-99.6%] vs 25.0% [95% CI, 5.5%-57.2%]; *P *=* *.02) breasts. However, sensitivity of mammography or ultrasonography alone did not exceed 80% across all breast densities in the 2 groups. Compared with the control group, specificity was significantly lower in the intervention group (91.8% [95% CI, 91.2%-92.3%] vs 86.8% [95% CI, 86.2%-87.5%]; *P* < .001). Recall rates (13.8% [95% CI, 13.1%-14.5%] vs 8.6% [95% CI, 8.0%-9.1%]; *P* < .001) and biopsy rates (5.5% [95% CI, 5.1%-6.0%] vs 2.1% [95% CI, 1.8%-2.4%]; *P* < .001) were significantly higher in the intervention group than the control group.

**Conclusions and Relevance:**

In this secondary analysis of a randomized clinical trial, screening mammography alone demonstrated low sensitivity, whereas adjunctive ultrasonography was associated with increased sensitivity. These findings suggest that adjunctive ultrasonography has the potential to improve detection of early-stage and invasive cancers across both dense and nondense breasts. Supplemental ultrasonography should be considered as an appropriate imaging modality for breast cancer screening in asymptomatic women aged 40 to 49 years regardless of breast density.

**Trial Registration:**

NIPH Clinical Trial Identifier: UMIN000000757

## Key Points

**Question:**

Does the performance of adjunctive ultrasonography for breast cancer detection among women undergoing screening mammography change according to breast tissue density?

**Findings:**

In this secondary analysis of a randomized clinical trial, screening mammography alone demonstrated low sensitivity, whereas adjunctive ultrasonography improved sensitivity both in dense and nondense breasts.

**Meaning:**

These findings suggest that adjunctive ultrasonography has the potential to improve the detection of early-stage and invasive cancers across both dense and nondense breasts and to mitigate the flaws of mammographic screening.

## Teaser

This secondary analysis of a randomized clinical trial evaluates the performance of adjunctive ultrasonography for breast cancer screening in asymptomatic women aged 40 to 49 years undergoing screening mammography according to differences in breast density.

## Introduction

Globally, breast cancer is the most frequently diagnosed cancer and is the leading cause of cancer-related death in women.^1^ In Japan, breast cancer is also the leading cancer in women, with breast cancer incidence rates peaking among women aged 45 to 49 years.^2^ Mammography is the only screening modality that has been shown to be associated with reduced deaths caused by breast cancer. However, the sensitivity of mammography is variable and ranges from as high as 80% to 98% in women with fatty breast tissue to as low as 30% to 48% in women with dense breast tissue.^3,4^ Because of the limitations of mammography and increase in breast cancer awareness, a few study groups have investigated the performance characteristics of supplementary screening tools, including breast ultrasonography, tomosynthesis, and magnetic resonance imaging.^5,6,7,8^

Breast density has been shown to be independently associated with increased risk of the incidence of and mortality attributable to breast cancer in younger women compared with older women,^9,10^ with increased risk of interval cancers between screening.^6,11,12^ Multiple studies^6,11,13^ have demonstrated that supplemental screening using ultrasonography generates an incremental cancer detection rate at the expense of lower specificity and lower positive predictive values. However, because most studies have focused on women at high risk^3,6,7^ or those with dense breast tissue but negative mammography findings,^3,8,14,15^ the performance of ultrasonography as an adjunct to mammography according to differences in breast density classification or among women at average risk remains unknown.^4,11,13,16^ Consequently, the effect of supplemental screening on breast cancer outcomes is still unclear.^17^

To our knowledge, the Japan Strategic Anti-cancer Randomized Trial (J-START) is the only multicenter randomized clinical trial (RCT) to date to directly compare adjunctive ultrasonography with standard mammography to screen asymptomatic women aged 40 to 49 years.^18,19,20^ The primary analysis in the original report^18^ revealed that sensitivity was significantly higher in the intervention group (mammography with ultrasonography) than in the control group (mammography alone), whereas specificity was significantly lower. More cancers were detected in the intervention group than in the control group and were more frequently stages 0 and I. Furthermore, there was a significant reduction in interval cancers.^20^ However, results according to breast density were not described in detail,^21,22^ and further studies investigating specific breast density groups have been solicited.^21,23^ This secondary analysis of the J-START RCT aimed to address issues related to performance of each modality according to differences in breast density.

## Methods

### Study Design and Participants

The J-START research design of has been published in detail elsewhere.^18,19,20^ Participants were randomly assigned in a 1:1 ratio to undergo screening by either mammography plus ultrasonography (intervention group) or mammography alone (control group), with or without clinical breast examination, once a year for 2 years.

Following a planned protocol (Supplement 1), randomization was centrally conducted by the Japan Clinical Research Support Unit, which is responsible for data management and support for trial operations and independently from Tohoku University. An independent data safety and monitoring board was established to monitor the progress of the trial, which met every 6 months. The study protocol was developed in accordance with the principles of the Declaration of Helsinki. Ethics guidelines for clinical studies issued by the Ministry of Education, Culture, Sports, Science and Technology and the Ministry of Health, Labor and Welfare of Japan were followed. Ethics approval was granted by Tohoku University School of Medicine Research Ethics Committee and the Japan Cancer Society. Written informed consent was obtained from all participants. This study follows the Consolidated Standards of Reporting Trials (CONSORT) reporting guideline for RCTs.

Participants who had a personal history of breast cancer, including in situ cancer, or other cancers in the previous 5 years, or life expectancy of no more than 5 years, were ineligible for the study. Between July 2007 and March 2011, 76 119 asymptomatic women aged 40 to 49 years were enrolled from 42 study sites in 23 of 47 prefectures in Japan. In this study, we used cases enrolled from the screening center in Miyagi prefecture, because we could examine and confirm the breast density.

### Screening Method and Assessment

Standard mammography and ultrasonography techniques were used at all participating facilities. The physicians and technicians involved in J-START completed a 2-day, 16-hour education program for the standardization of ultrasonography screening for breast cancer.^24^ Handheld ultrasonography was performed by a technician or by a physician, and later, the ultrasonography image was interpreted by another physician. The ultrasonography examination was performed, on average, in 10 minutes. Mammography acquisition, practice, apparatus, and interpretation were certified by the Japan Central Organization on Quality Assurance of Breast Cancer Screening. Ultrasonography acquisition, practice, apparatus, and interpretation were certified by the Japan Association of Breast and Thyroid Sonology.^18^

Mammography, ultrasonography, and clinical breast examination were performed and interpreted independently from one another, and images were interpreted with double reading by 2 authorized physicians.^18,19^ The independently assessed findings of mammography, ultrasonography, and clinical examination were classified into 5 categories that are used locally and internationally: 1, no findings; 2, benign; 3, probably benign but further assessment needed; 4, probably malignant; and 5, malignant.^19,25,26^ Further assessment was considered test-positive if any of the findings were categorized as 3 or higher.

For the purpose of this secondary analysis, which was conducted from March to September 2019, 3 expert physicians revaluated mammographic density of the first screening image. All information on screening results, medical interview record, and follow-up data were blinded. By use of the fifth edition of the Breast Imaging Reporting and Data System (BI-RADS),^27^ we classified visual judgment data as follows: almost entirely fatty; scattered areas of fibroglandular density; heterogeneously dense, which may obscure detection of small masses; and extremely dense, which lowers the sensitivity of mammography screening. In the present article, fatty and scattered are referred to as nondense, and heterogeneously dense and extremely dense are referred to as dense.

### Follow-up

Breast cancers were ascertained by diagnostic assessment of first and second screening results or by a mail survey questionnaire to patients who did not attend the second screening. If data were not available, resident registers were used to identify the patient’s living status. In addition, date linkage with both hospital discharge records and cancer registry databases was used to identify breast cancer diagnosis information. Miyagi prefecture has a sophisticated system of local registration for cancer that contains data on virtually all patients with breast cancer in Miyagi prefecture. Therefore, it was possible to identify interval cancers exactly, and follow-up for this study was updated in February 2020.

Screen-detected breast cancers were defined as those categorized as categories 3 to 5 at the first-round screening, and interval cancers were defined as those diagnosed between the first round and the second round of screening for which the initial category had been 1 or 2. Recall was defined as the need for any additional diagnostic testing after screening, including imaging and/or biopsy. The clinical stage and histopathological data were classified by the seventh edition of the TNM classification system.^28^ The outcome definitions and measure methods were prespecified before data release. Breast cancers diagnosed by the second-round screening were not counted for this study analysis.

### Statistical Analysis

The preliminary sample size determination in J-START has been published in detail elsewhere.^19,20^ We used data from the participants at screening center in Miyagi prefecture. The special feature of the present study was not only to clarify differences between the intervention and control groups, but also differences in breast density composition. Sensitivity, specificity, recall rates, cancer detection rates, interval cancer rates, biopsy rates, and characteristics of screen-detected and interval breast cancers of the first-round screening were examined.

All outcomes were analyzed according to the intention-to-treat principle. Performance outcomes were assessed with generalized estimating equations with an exchangeable working correlation matrix and robust SEs. Fisher exact test was used to detect significant differences in the clinical stage and histological findings between cancers detected by ultrasonography and mammography within the intervention group. All tests were 2-sided, and significance was set at *P* < .05. All statistical analyses were done with SAS statistical software version 9. 4 (SAS Institute). Data analysis was performed from February to March 2020.

## Results

### Participant Characteristics

The baseline characteristics of study participants are shown in Table 1. Of 72 998 asymptomatic women aged 40 to 49 years enrolled in J-START, 19 213 women (9705 in the intervention group and 9508 in the control group) were analyzed in this study because they were residents in Miyagi Prefecture where the cancer registry had been established.

**Table 1.** Baseline Characteristics of Study Participants

Characteristic	Participants, No. (%) (N = 19 213)^a^					
	Total intervention group (n = 9705)	Total control group (n = 9508)	Intervention group by breast density		Control group by breast density	
			Dense (n = 5797)	Nondense (n = 3908)^b^	Dense (n = 5593)^b^	Nondense (n = 3915)^b^
Age, mean (SD), y	44.5 (2.9)	44.6 (2.9)	44.5 (2.9)	44.7 (2.9)	44.5 (2.9)	44.6 (2.9)
Ever undergo breast cancer screening						
No	1687 (17.4)	1895 (19.9)	995 (17.2)	692 (17.7)	1094 (19.6)	801 (20.5)
Yes	8018 (82.6)	7613 (80.1)	4802 (82.8)	3216 (82.3)	4499 (80.4)	3114 (79.5)
Time since most recent breast cancer screening, mo						
<12	716 (8.9)	681 (9.0)	441 (9.2)	275 (8.6)	424 (9.4)	257 (8.3)
12-24	2942 (36.7)	2773 (36.4)	1759 (36.6)	1183 (36.8)	1620 (36.0)	1153 (37.0)
25-36	2916 (36.4)	2806 (36.9)	1710 (35.6)	1206 (37.5)	1667 (37.1)	1139 (36.6)
>36	1391 (17.4)	1308 (17.2)	859 (17.9)	532 (16.5)	762 (16.9)	546 (17.5)
Unknown or data missing	53 (0.7)	45 (0.6)	33 (0.7)	20 (0.6)	26 (0.6)	19 (0.6)
Method of most recent breast cancer screening						
Mammography						
No	2342 (29.2)	2191 (28.8)	1419 (29.6)	923 (28.7)	1308 (29.1)	883 (28.4)
Yes	5676 (70.8)	5422 (71.2)	3383 (70.5)	2293 (71.3)	3191 (70.9)	2231 (71.6)
Ultrasonography						
No	6406 (79.9)	6221 (81.7)	3800 (79.1)	2606 (81.0)	3647 (81.1)	2574 (82.7)
Yes	1612 (20.1)	1392 (18.3)	1002 (20.9)	610 (19.0)	852 (18.9)	540 (17.3)
Clinical breast examination						
No	356 (4.4)	332 (4.4)	215 (4.5)	141 (4.4)	194 (4.3)	138 (4.4)
Yes	7662 (95.6)	7281 (95.6)	4587 (95.5)	3075 (95.6)	4305 (95.7)	2976 (95.6)
Age at menarche, y						
≤9	25 (0.3)	25 (0.3)	12 (0.2)	13 (0.3)	9 (0.2)	16 (0.4)
10-15	9559 (98.5)	9383 (98.7)	5709 (98.5)	3850 (98.5)	5517 (98.6)	3866 (98.8)
≥16	121 (1.3)	100 (1.1)	76 (1.3)	45 (1.2)	67 (1.2)	33 (0.8)
Menopausal status						
Premenopausal	7353 (75.8)	7285 (76.6)	4502 (77.7)	2851 (73.0)	4399 (78.7)	2886 (73.7)
Perimenopausal	1699 (17.5)	1615 (17.0)	948 (16.4)	751 (19.2)	876 (15.7)	739 (18.9)
Postmenopausal	652 (6.7)	603 (6.3)	347 (6.0)	305 (7.8)	315 (5.6)	288 (7.4)
Unknown or data missing	1 (<0.1)	5 (<0.1)	0	1 (<0.1)	3 (<0.1)	2 (<0.1)
Pregnancies, No.						
0	813 (8.4)	742 (7.8)	606 (10.5)	207 (5.3)	520 (9.3)	222 (5.7)
1	1192 (12.3)	1170 (12.3)	768 (13.3)	424 (36.5)	762 (13.6)	408 (10.4)
2	3659 (37.7)	3603 (37.9)	2233 (38.5)	1426 (36.5)	2154 (38.5)	1449 (37.0)
3-4	3425 (35.3)	3328 (35.0)	1875 (38.5)	1550 (39.7)	1757 (31.4)	1571 (40.1)
5-10	372 (3.8)	400 (4.2)	159 (2.7)	213 (5.5)	212 (3.8)	188 (4.8)
Unknown or data missing	244 (2.5)	265 (2.8)	156 (2.7)	88 (2.3)	188 (3.4)	77 (2.0)
Pregnancies delivered, No.						
Nulliparous	130 (1.5)	124 (1.5)	96 (1.9)	34 (0.9)	85 (1.7)	39 (1.1)
1	1427 (16.5)	1423 (16.7)	924 (18.4)	503 (13.9)	929 (19.0)	494 (13.7)
2	4499 (52.0)	4448 (52.3)	2690 (18.4)	1809 (50.1)	2625 (53.7)	1823 (50.4)
3	2270 (26.3)	2202 (25.9)	1191 (23.7)	1079 (29.9)	1113 (22.8)	1089 (30.1)
4-8	291 (3.4)	257 (3.0)	118 (2.3)	173 (4.8)	105 (2.2)	152 (4.2)
Unknown or data missing	31 (0.4)	47 (0.6)	16 (2.3)	15 (0.4)	28 (0.6)	19 (0.5)
Age at first parturition, y						
<20	102 (1.2)	80 (0.9)	58 (1.2)	44 (1.2)	44 (0.9)	36 (1.0)
20-24	1626 (18.8)	1470 (17.3)	897 (17.8)	729 (20.2)	783 (16.0)	687 (19.0)
25-29	3245 (37.5)	3184 (37.5)	1901 (37.8)	1344 (37.2)	1811 (37.1)	1373 (38.0)
30-39	1882 (21.8)	2022 (23.8)	1132 (22.5)	750 (20.8)	1239 (25.4)	783 (21.7)
40-49	48 (0.6)	47 (0.6)	36 (0.7)	12 (0.3)	27 (0.6)	20 (0.6)
Unknown or data missing	1745 (20.2)	1698 (20.0)	1011 (20.1)	734 (20.3)	981 (20.1)	717 (19.8)
Ever breastfed children						
Yes	7754 (90.9)	7570 (90.4)	4507 (91.1)	3247 (90.6)	4338 (90.4)	3232 (90.4)
No	758 (8.9)	792 (9.5)	428 (8.7)	330 (9.2)	455 (9.5)	337 (9.4)
Unknown or data missing	21 (0.3)	16 (0.2)	15 (0.3)	6 (0.2)	8 (0.2)	8 (0.2)
First-degree female relatives with breast cancer, No.						
0	9214 (94.9)	9024 (94.9)	5503 (94.9)	3711 (95.0)	5299 (94.7)	3725 (95.2)
1	485 (5.0)	477 (5.0)	291 (5.0)	194 (5.0)	289 (5.2)	188 (4.8)
>1	6 (<0.1)	7 (<0.1)	3 (<0.1)	3 (<0.1)	5 (<0.1)	2 (<0.1)
Ever had breast surgery	227 (1.2)	214 (1.1)	170 (1.8)	57 (0.6)	140 (1.5)	74 (0.8)
Ever had benign neoplasm	154 (0.8)	143 (0.7)	121 (1.3)	33 (0.3)	97 (1.0)	46 (0.5)
Ever had breast inflammation	71 (0.4)	70 (0.4)	46 (0.5)	25 (0.3)	45 (0.5)	25 (0.3)

^a^ Percentages might not total 100% because of rounding.

^b^ The 2 least dense categories (almost entirely fatty and scattered fibroglandular tissues) are referred to as nondense, conventionally, the 2 most dense categories (heterogeneously dense and extremely dense) are referred to as dense.

An overview of participant flow is shown in the eFigure in Supplement 2. A total of 19 280 participants were enrolled and randomly allocated either study arms. Eligibility was assessed for inclusion in the analyses; 26 participants in the intervention group and 41 participants in the control group were excluded because of ineligibility and were withdrawn. Of 19 213 participants, 11 390 (59.3%) were categorized as having dense breast tissue (ie, heterogeneously or extremely dense) (eTable in Supplement 2).

The mean (SD) age of participants was 44.5 (2.8) years (Table 1). A total of 975 participants (5.0%) reported a history of breast cancer in first-degree female relatives, and 297 participants (1.5%) reported having ever had benign breast diseases. There were no differences in demographic characteristics or risk factors between the intervention and control groups. On the other hand, the percentages of participants who had never been pregnant were significantly higher among women with dense breasts than among women with nondense breasts in both the intervention group (10.5% [95% CI, 9.9%-11.6%] vs 5.3% [95% CI, 4.7%-6.1%]; *P* < .001) and control group (9.3% [95% CI, 8.8%-10.4%] vs 5.7% [95% CI, 5.1%-6.5%] *P* < .001).

### Screening Performance

Table 2 and Table 3 summarize the screening performance. In 19 213 women, 130 cancers were found. More screen-detected cancers were found in the intervention group than in the control group (68 cancers [7.0 cancers per 1000 screenings; 95% CI, 5.3 to 8.7 cancers per 1000 screenings] vs 38 cancers [4.0 cancers per 1000 screenings; [95% CI, 2.7 to 5.3 cancers per 1000 screenings]; *P* = .004). Among women with dense breasts, there were more screen-detected cancer in the intervention group than the control group (41 cancers [7.1 cancers per 1000 screenings; 95% CI, 4.9 to 9.2 cancers per 1000 screenings] vs 24 cancers [4.3 cancers per 1000 screenings; 95% CI, 2.6 to 6.0 cancers per 1000 screenings]; *P* = .04). A similar tendency is seen in women with nondense breasts (27 cancers [6.9 cancers per 1000 screenings; 95% CI, 4.3 to 9.5 cancers per 1000 screenings] in the intervention group vs 14 cancers [3.6 cancers per 1000 screenings; 95% CI, 1.7 to 5.4 cancers per 1000 screenings] in the control group; *P* = .04). Five interval cancers (0.5 cancers per 1000 screenings; 95% CI, 0.1 to 1.0 cancers per 1000 screenings) were detected in the intervention group compared with 19 (2.0 cancers per 1000 screenings; 95% CI, 1.1 to 2.9 cancers per 1000 screenings) in the control group (*P* = .004), with a significant difference between the 2 groups among women with dense breasts (3 cancers [0.5 cancers per 1000 screenings; 95% CI, −0.1 to 1.1 cancers per 1000 screenings] vs 10 cancers [1.8 cancers per 1000 screenings; 95% CI, 0.7 to 2.9 cancers per 1000 screenings]; *P* = .04) as well as women with nondense breasts (2 cancers [0.5 cancers per 1000 screenings; 95% CI, −0.2 to 1.2 cancers per 1000 screenings] vs 9 cancers [2.3 cancers per 1000 screenings; 95% CI, 0.8 to 3.8 cancers per 1000 screenings]; *P* = .03). Sensitivity in the intervention group was higher than that in the control group (93.2% [95% CI, 87.4% to 99.0%] vs 66.7% [95% CI, 54.4% to 78.9%]; *P* < .001) for both dense breasts and nondense breasts. In contrast, specificity was significantly lower in the intervention group than the control group (86.8% [95% CI, 86.2% to 87.5%] vs 91.8% [95% CI, 91.2% to 92.3%]; *P* < .001) regardless of breast tissue density (Table 2).

**Table 2.** Performance According to Breast Density Category

Variable	Total participants (N = 19 213)			Dense breasts^a^			Nondense breasts^a^		
	Intervention group (n = 9705)	Control group (n = 9508)	*P* value	Intervention group (n = 5797)	Control group (n = 5593)	*P* value	Intervention group (n = 3908)	Control group (n = 3915)	*P* value
Screen-detected cancers									
No. of cancers/total No.	68/9705	38/9508	.004	41/5797	24/5593	.04	27/3908	14/3915	.04
No. of cancers per 1000 screenings (95% CI)	7 (5.3 to 8.7)	4 (2.7 to 5.3)		7.1 (4.9 to 9.2)	4.3 (2.6 to 6.0)		6.9 (4.3 to 9.5)	3.6 (1.7 to 5.4)	
Interval cancers									
No. of cancers/total No.	5/9705	19/9508	.004	3/5797	10/5593	.04	2/3908	9/3915	.03
No. of cancers per 1000 screenings (95% CI)	0.5 (0.1 to 1.0)	2.0 (1.1 to 2.9)		0.5 (−0.1 to 1.1)	1.8 (0.7 to 2.9)		0.5 (−0.2 to 1.2)	2.3 (0.8 to 3.8)	
Sensitivity, % (95% CI)	93.2 (87.4 to 99.0)	66.7 (54.4 to 78.9)	<.001	93.2 (85.7 to 100)	70.6 (55.3 to 85.9)	<.001	93.1 (83.9 to 102.3)	60.9 (40.9 to 80.8)	<.001
Specificity, % (95% CI)	86.8 (86.2 to 87.5)	91.8 (91.2 to 92.3)	<.001	85.4 (84.5 to 86.3)	91.7 (91.0 to 92.4)	<.001	89.0 (88.0 to 90.0)	91.9 (91.1 to 92.8)	<.001

^a^ The 2 least dense categories (almost entirely fatty and scattered fibroglandular tissues) are referred to as nondense, and the 2 most dense categories (heterogeneously dense and extremely dense) are referred to as dense.

**Table 3.** Sensitivity of Each Modality According to Breast Densities and Study Groups

Group and modality	Cancers, No. (%) [95% CI]		*P* value
	Dense breasts^a^	Nondense breasts^a^	
Intervention group (n = 9705)			
Screen-detected cancers (n = 68)			
Mammography positive, ultrasonography positive or negative, CBE positive or negative	24 (54.6) [39.8-69.3]	20 (69.0) [52.1-85.8]	.20
Mammography positive or negative, ultrasonography positive, CBE positive or negative	29 (65.9) [51.9-79.9]	15 (51.7) [33.5-69.9]	.22
Mammography positive, ultrasonography positive, CBE positive or negative	12 (27.3) [14.1-40.3]	8 (27.6) [11.3-43.9]	.98
Mammography positive or negative, US positive or negative, CBE positive	10 (22.7) [10.3-35.1]	10 (34.5) [17.2-51.8]	.28
Only mammography positive	12 (27.3) [14.1-40.3]	12 (41.4) [23.5-59.3]	.21
Only ultrasonography positive	17 (38.6) [24.3-53.0]	7 (24.1) [8.6-39.7]	.18
Only CBE positive	0 (NA)	0 (NA)	NA
Any positive	41 (93.2) [85.7-100.0]	27 (93.1) [83.9-102.3]	.98
Interval cancers (n = 5), all modalities negative	3 (NA)	2 (NA)	NA
Control group (n = 9508)			
Screen-detected cancers (n = 38)			
Mammography positive, CBE positive or negative	22 (64.7) [48.6-80.8]	14 (60.9) [40.9-80.8]	.77
Mammography positive or negative, CBE positive	9 (26.5) [11.6-41.3]	6 (26.1) [8.1-44.0]	.97
Only mammography positive	15 (44.1) [27.3-60.8]	8 (34.8) [15.3-54.3]	.48
Only CBE positive	2 (NA)	0 (NA)	NA
Either mammography or CBE positive	24 (70.6) [55.3-85.9]	14 (60.9) [40.9-80.8]	.45
Interval cancers (n = 19), all modalities negative	10 (NA)	9 (NA)	NA

Abbreviations: CBE, clinical breast examination; NA, not applicable.

^a^ The 2 least dense categories (almost entirely fatty and scattered fibroglandular tissues) are referred to as nondense, and the 2 most dense categories (heterogeneously dense and extremely dense) are referred to as dense.

Table 3 summarizes sensitivity of each modality according to density. Neither modality exceeded 80% sensitivity alone (Table 3). In the intervention group, sensitivity of mammography was 69.0% (95% CI, 52.1%-85.8%) in nondense breasts and 54.6% (95% CI, 39.8%-69.3%) in dense breasts, whereas the sensitivity of mammography with adjunct ultrasonography was 93.1% (95% CI, 83.9%-102.3%) in nondense breasts and 93.2% (95% CI, 85.7%-100.0%) in dense breasts. The sensitivity of mammography was higher in women with dense breasts than those with nondense breasts (70.6% [95% CI, 55.3%-85.9%] vs 60.9% [95% CI, 40.9%-80.8%]).

The frequency of clinical stage 0 and I breast cancer was 85.4% in dense breasts and 88.9% in nondense breasts within the intervention group, whereas in the control group, the frequency was 79.2% for dense breasts and 64.3% for nondense breasts. The differences in stage between the 2 groups were similar for dense and nondense breasts (Table 4). Within the intervention group, the rate of invasive cancers detected by ultrasonography alone was significantly higher than that for mammography alone, in both dense breasts (82.4% [95% CI, 56.6%-96.2%] vs 41.7% [95% CI, 15.2%-72.3%]; *P = *.02) and nondense breasts (85.7% [95% CI, 42.1%-99.6%] vs 25% [95% CI, 5.5%-57.2%]; *P *= .02). In the control group, however, the clinical stage and each pathological finding of invasive cancers detected by mammography alone were not meaningfully different between dense and nondense breasts (Table 4).

**Table 4.** Clinical Stage and Histological Findings of Screen-Detected Cancers and Interval Cancers According to Breast Density

	Patients, No. (%)^a^								
	Intervention group					Control group			
	Screen-detected cancers (n = 68)				Interval cancers (n = 5)	Screen-detected cancers (n = 38)			Interval cancers (n = 19)
	Any modality positive (n = 68)	Only mammography positive (n = 24)	Only US positive (n = 24)	Only CBE positive (n = 0)		Either positive (n = 38)	Only mammography positive (n = 23)	Only CBE positive (n = 2)	
Extremely and heterogeneously dense^b^									
Breast cancers, No.	41	12	17	0	3	24	15	2	10
Clinical stage^c^									
0 and I	35 (85.4)	12 (100)	2 (76.5)	0	1 (33.3)	19 (79.2)	15 (100)	1 (50)	9 (90)
II or higher	6 (14.6)	0	4 (23.5)	0	2 (66.7)	5 (20.8)	0	1 (50)	1 (10)
Histopathological cancer type									
Noninvasive^d^	13 (31.7)	7 (58.3)	3 (17.7)	0	0	6 (25)	6 (40)	0	3 (30)
Invasive^e^	28 (68.3)	5 (41.7)	14 (82.4)	0	3 (100)	18 (75)	9 (60)	2 (100)	7 (70)
Size of invasive tumors on histological examination, mm									
<10	11 (39.3)	5 (100)	4 (28.6)	0	1 (33.3)	9 (50)	6 (66.7)	1 (50)	1 (14.3)
11-20	15 (53.6)	0	8 (57.1)	0	0	4 (22.2)	2 (22.2)	1 (50)	4 (57.1)
>20	2 (7.1)	0	2 (14.3)	0	2 (66.7)	4 (22.2)	0	0	0
Data missing	0	0	0	0	0	1 (5.6)	1 (11.1)	0	2 (28.6)
Node status of invasive cancers									
Negative	23 (82.1)	5 (100)	11 (78.6)	0	1 (33.3)	15 (83.3)	8 (88.9)	2 (100)	6 (85.7)
Positive	5 (17.9)	0	3 (21.4)	0	2 (66.7)	2 (11.1)	0	0	0
Data missing	0	0	0	0	0	1 (5.6)	1 (11.1)	0	1 (14.3)
Scattered fibroglandular tissue and almost entirely fatty^b^									
Breast cancers, No.	27	12	7	0	2	14	8	0	9
Clinical stage^c^									
0 and I	24 (88.9)	11 (91.7)	6 (86.7)	0	1 (50)	9 (64.3)	5 (62.5)	0	9 (100)
II or higher	3 (11.1)	1 (8.3)	1 (14.3)	0	1 (50)	5 (35.7)	3 (37.5)	0	0
Histopathological cancer type									
Noninvasive^d^	13 (48.2)	9 (75)	1 (14.3)	0	0	3 (21.4)	3 (37.5)	0	2 (22.2)
Invasive^e^	14 (51.9)	3 (25)	6 (85.7)	0	2 (100)	11 (78.6)	5 (62.5)	0	7 (77.8)
Size of invasive tumors on histological examination, mm									
≤10	5 (35.7)	0	4 (66.7)	0	1 (50)	4 (36.4)	3 (60)	0	2 (28.6)
11-20	9 (64.3)	3 (100)	2 (33.3)	0	0	5 (45.5)	1 (20)	0	4 (57.1)
>20	0	0	0	0	1 (50)	2 (18.2)	1 (20)	0	0
Data missing	0	0	0	0	0	0	0	0	1 (14.3)
Node status of invasive cancers									
Negative	11 (78.6)	2 (66.7)	5 (83.3)	0	2 (100)	6 (54.6)	3 (60)	0	6 (85.7)
Positive	3 (21.4)	1 (33.3)	1 (16.7)	0	0	5 (45.5)	2 (40)	0	0
Data missing	0	0	0	0	0	0	0	0	1 (14.3)

Abbreviations: CBE, clinical breast examination; US, ultrasonography.

^a^ Percentages might not total 100% because of rounding.

^b^ No cancer was found in the category of almost entirely fatty.

^c^ Based on the *International Statistical Classification of Diseases and Related Health Problems, Tenth Revision*.

^d^ Includes ductal carcinoma in situ and lobular carcinoma in situ.

^e^ Includes invasive ductal carcinoma and special type.

Data of screening recalls and biopsy are presented in Table 5. Of 2147 participants who were recalled, 734 underwent biopsies. The recall rates (13.8% [95% CI, 13.1%-14.5%] vs 8.6% [95% CI, 8.0%-9.1%]; *P* < .001) and biopsy rates (5.5% [95% CI, 5.1%-6.0%] vs 2.1% [95% CI, 1.8%-2.4%]; *P* < .001) were significantly greater in the intervention group vs the control group. Recall rates for mammography alone were similar in the 2 groups regardless of breast density. In the intervention group, the recall rate by ultrasonography alone was higher for women with dense breasts than for women with nondense breasts (7.0% vs 3.9%), and the same was true for the biopsy rate (4.4% vs 2.6%).

**Table 5.** Recall Rate and Biopsy Rate of Each Modality According to Study Group

Variable	Participants, No. (%)					
	Intervention group			Control group		
	Total (n = 9705)	Dense breasts^a^		Total (n = 9508)	Dense breasts^a^	
		Yes (n = 5797)	No (n = 3908)		Yes (n = 5593)	No (n = 3915)
Recalled after first-round screening						
Any modality positive	1334 (13.8)	880 (15.2)	454 (11.6)	813 (8.6)	485 (8.7)	328 (8.4)
Only mammography positive	606 (6.2)	356 (6.1)	250 (6.4)	663 (7.0)	374 (6.7)	290 (7.4)
Only ultrasonography positive	558 (5.8)	404 (7.0)	154 (3.9)	NA	NA	NA
Only CBE positive	59 (0.6)	44 (0.8)	15 (0.4)	85 (0.9)	69 (1.2)	16 (0.4)
Biopsy rate^b^						
Biopsy done	538 (5.5)	360 (6.2)	178 (4.6)	196 (2.1)	127 (2.3)	69 (1.8)
Only mammography positive	107 (1.1)	56 (1.0)	51 (1.3)	128 (1.4)	77 (1.4)	51 (1.3)
Only ultrasonography positive	355 (3.7)	255 (4.4)	100 (2.6)	NA	NA	NA
Only CBE positive	7 (<0.1)	4 (<0.1)	3 (<0.1)	27 (0.3)	22 (0.4)	5 (<0.1)

Abbreviations: CBE, clinical breast examination; NA, not applicable.

^a^ The 2 least dense categories (almost entirely fatty and scattered fibroglandular tissues) are referred to as nondense, and the 2 most dense categories (heterogeneously dense and extremely dense) are referred to as dense.

^b^ Indicates a need for biopsy on first-round screening. When clinically indicated, participants might have undergone 2 or more types of biopsy.

## Discussion

To our knowledge, J-START is the first large-scale RCT to assess the performance of ultrasonography screening in combination with mammography for breast cancer in women aged 40 to 49 years with average risk. Now, the question is raised as to whether adjunctive ultrasonography improves the balance of breast cancer screening in women with different breast densities. In the present secondary analysis, we further evaluated the performance of each screening modality (ie, mammography and ultrasonography) according to breast density, which has been shown to be a factor independently associated with increased risk of breast cancer across age group.^9^ Heterogeneity of tissue density is associated with not only increased cancer risk but also complications of mammographic interpretation; even small amounts of dense tissue can mask cancer. The fifth edition of the BI-RADS lexicon for breast density^27^ recommends that breast imagers assign breast composition descriptors that better convey whether there are dense areas of tissue that could mask or obscure cancer. In this respect, a major strength of this study is that we used the fifth edition of the BI-RADS lexicon to assess breast density and conducted double readings to reduce variation, which provided reproducible estimates and verification of density assessment quality. With awareness of these issues (breast density and lower screening sensitivity of mammography) as the starting point, we analyzed the performance of adjunctive ultrasonography compared with mammography in breasts with different densities. The principal findings between the intervention and control groups were consistent with those of the J-START,^20^ as adjunct ultrasonography to mammography exhibited higher sensitivity and lower specificity than mammography alone, with more cancers detected, more cancers that were stages 0 and I, and lower numbers of interval cancers than in the control group.^20^ Adjunct ultrasonography improved the sensitivity and detection rate of small, early-stage (stage 0 and stage I) and invasive cancers, consistent with other studies.^3,7,11^

In addition, this study revealed that adjunctive ultrasonography improved the detection of early invasive cancers not only in dense breasts but also in nondense breasts. Moreover, we found ultrasonography to be potentially superior to mammography in detecting early and node-negative invasive cancers in both dense and nondense breasts.

It should be noted that sensitivity of mammography alone and that of ultrasonography alone were both lower than the sensitivity of the combination of these modalities. In this study, the sensitivity of mammography was higher in women with dense breasts than those with nondense breasts (70.6% vs 60.9%). One of the explanations is that the sensitivity of mammography did not depend on breast density, because even nondense tissue might hide or mask cancer in women aged 40 to 49 years. However, sensitivity was improved when ultrasonography was used as an adjunct to mammography, suggesting that adjunctive ultrasonography has an advantage in breast cancer screening for young women regardless of dense breasts. Taking these results into account, we can conclude that breast density should not be the sole criterion for deciding whether supplemental imaging is justified. Because of the limitations of mammography in breast density, studies have investigated performance of supplementary screening tools, including tomosynthesis and magnetic resonance imaging.^5,6,7,8^ Tomosynthesis is unlikely to be an optimal solution, and magnetic resonance imaging is expensive and not easy to access for screening.

With regard to the harms associated with breast cancer screening, supplemental ultrasonography had lower specificity and higher recall and biopsy rates than mammography alone, which are major limitations of screening ultrasonography.^14,29^ Ultrasonography and mammography findings were interpreted independently according to the J-START protocol, which consequently increased recall and biopsy rates and decreased specificity. In addition, ultrasonography-guided histological examination using core-needle biopsy is easy to perform and more accurate for diagnosing the lesion, which suggests that it is the main reason for the increased biopsy rate. A study^30^ of the European mammography screening programs showed overall further assessment rates to be 9.3% and 4.0% for initial and subsequent mammography screening tests, respectively. The overall rates of needle biopsy rates were 2.2% and 1.1%, respectively.^30^ A previous study^31^ in Japan reported that recall rate of mammography screening among 33 924 women in their 40s was 9.9%. The recall rate of the first round in J-START^31^ was 8.8% in the control group (mammography only), which is within the accepted range.

### Limitations

The sensitivity and specificity in this study were calculated with the data from the first-round screening. Our findings cannot be extended beyond the first round, because characteristics of breast cancer would differ between the first and later rounds of screening.^7,12^ Except for women at increased and high risk, estimates indicate a nearly 40% breast cancer mortality reduction when screening women annually starting at age 40 years.^32^ Improving cancer detection in younger and middle-aged women is crucial for increasing the effectiveness of breast cancer screening.^23^ To evaluate screening benefits avoiding lead time bias, and to determine balance of benefits and harms, further investigation providing hard evidence about the contributions of adjunctive ultrasonography screening to breast cancer mortality is necessary.

## Conclusions

In this secondary analysis of an RCT, adjunctive ultrasonography had good screening balance with mammography regardless of breast density, detecting early-stage and invasive malignant lesions for asymptomatic women with average risk of breast cancer. Thus, adjunctive ultrasonography should be considered as an optimal solution in young women with average risk.

## Notes

Supplement 1.
Trial Protocol and Statistical Analysis Plan

Supplement 2. **eFigure.** Trial Profile **eTable.** Distribution of Mammography Density Based on 5th Edition BI-RADS Density Categories

Supplement 3.
Data Sharing Statement
