# Supplementary material for: Evaluation of Adjunctive Ultrasonography for Breast Cancer Detection Among Women Aged 40-49 Years With Varying Breast Density Undergoing Screening Mammography: A Secondary Analysis of a Randomized Clinical Trial
Source: JAMA Netw Open. 2021 Aug 18;4(8):e2121505. doi: 10.1001/jamanetworkopen.2021.21505 (PMC8374606; doi:10.1001/jamanetworkopen.2021.21505)
Supplement: Supplement 1. — Trial Protocol and Statistical Analysis Plan [file jamanetwopen-e2121505-s001.pdf]

Strategic Research on Countermeasures against Cancer (Topic 1), the Third Comprehensive Anti-Cancer Strategic Research Program, Ministry of Health, Labour and Welfare, for Fiscal 2007

Protocol -- Comparative Study to Evaluate the Usefulness of Ultrasonography as a Means of Breast Cancer Screening

Ver.6.0

(January 18, 2019)

Contents

Overview 4
Background and outline 4
Objectives: 5
Areas covered 5
Method of intervention 5
Endpoints 5
Study period 5
Research leader 5
1. Background 6
2. Objectives 8
3. Study design 8
4. Criteria for selection of subjects 11
4.1. Inclusion criteria 11
4.2. Exclusion criteria 11
4.3. Population for subject recruitment 11
5. Organization and facilities participating in the study 11
6. Screening methods 12
6.1 Ultrasonography-combined screening (intervention group) 12
6.1.1. Screening with mammography 12
6.1.2. Screening with ultrasonography 13
6.2. Ultrasonography uncombined screening (non-intervention group) 13
6.2.1. Screening mammography 13
6.3.Clinical breast examination 14
7. Study schedule 14
8. Survey and observation 15
8.1. At the time of enrollment 15
8.2. Information related to screening results 15
8.3. Information related to the individuals receiving a detailed test 17
8.4. Screening results report to subjects 17
8.5. Follow-up 17
9. Follow-up method 18
10. Safety management 18
10.1. Duties of the responsible person of each participating organization 19
10.2. Duties of Research Leader 19

11. Statistical analyses 20
11.1. Major statistical analyses 20
11.2. Follow-up 20
12. Study period 20
13. Ethical consideration: Informed consent 20
13.1. Use of individual information 21
13.2. Explanation and consent 21
13.3. Authorization of the protocol 22
13.4. Compensation related to the study 22
14. Discontinuation of the study 22
15. Storage, disposal or deletion of records 22
16. Data management and monitoring 22
16.1. Institutional data center 23
16.2. Central Data Center 23
17. Review and modification of the protocol 25
18. Additional compound study 25
19. Publication of study outcomes 25
20. Study participants other than subjects and the study organization 26
20.1.Study participants 26
20.2. Study organization and roles 26
21. References
Appendices: Summary of protocol changes

Overview

Background and outline

Breast cancer is the most frequent type of cancer for females. The death rate from breast cancer is higher than that from any other cancer for the age group 30-69. Among others, the age group 40-49 has recently been showing a marked increase in the incidence of breast cancer. Therefore, there is an urgent need to countermeasures for reducing the death rate from breast cancer among women in this age group.

Mammography, which is a global standard of breast cancer screening, is low in accuracy when used for breasts with high mammary gland density. High density breasts are often seen at ages less than 50 and more frequent among Japanese women than among Western women. Ultrasonography has high accuracy when used for checking breasts with high mammary gland density. Attempts to use ultrasonography as a means of breast cancer screening have already begun to be made. However, neither the specifications of devices for breast ultrasonography nor its procedure and image reading techniques have yet been standardized. Furthermore, the accuracy and usefulness of ultrasonography as a means of breast cancer screening have not been endorsed.

This study is designed to compare the effect in reducing the death rate from breast cancer between two groups of women, i.e., between women screened with standardized ultrasonography + mammography and women screened with mammography without ultrasonography.

Standardization and spread of ultrasonographic breast cancer screening

↓

Subjects : Women aged 40-49 (60,000 women/group)
Comparison : Mammography + Ultrasonography Group vs Mammography Group
Outcome : Accuracy (sensitivity, specificity), Cumulative incidence of advanced breast cancer

↓

Reduction in death rate from breast cancer

Objectives:

Evaluation of the accuracy and usefulness of ultrasonic screening of breast cancer.

Areas covered

Facilities and organization (openly invited for participation) providing breast cancer screening in Japan

Method of intervention

About 120,000 women aged 40-49 are divided into the intervention group (screened with mammography + ultrasonography) and the non-intervention group (screened without ultrasonography).

Endpoints

Primary endpoint: Inter-group comparison of sensitivity, specificity and detection rate.

Secondary endpoint: Inter-group comparison of the cumulative incidence of advanced breast cancer during the follow-up period

Study period

July 2007 through March 2013

Research leader

Noriaki Ohuchi, Professor, Department of Surgical Oncology, Tohoku University Graduate School of Medicine

1. Background

Since 1994, the incidence of breast cancer has been higher than that of any other female cancer. Among other, the incidence of this cancer in females aged between 40 and 49 is expected to further rise from now on following changes in the lifestyle of Japanese people [1,2,3]. Premenopausal breast cancer, developing at age 40-49, has been sharply increasing in recent years, and this cancer is related to estrogen, one of the female hormones. It is said that women with lower age of menarche, no history of delivery and delay in menopause are more likely to develop breast cancer because of prolonged exposure to estrogen [3]. However, primary prophylaxis, designed to remove these factors, is not realistic. Under the current trend for less child delivery by individual women, a valid means of preventing death from breast cancer is secondary prophylaxis designed to facilitate early detection of breast cancer, i.e., development, spread, utilization and improvement of a valid method of breast cancer screening.

Breast cancer screening with mammography has been shown in multiple randomized comparative studies in Western countries to be useful in reducing the death rate from breast cancer among women aged over 50, and has been adopted as a global standard. However, the usefulness of this way of screening in reducing the death from breast cancer is lower for women aged 40-49 than for women aged over 50 [4,5,6]. Furthermore, mammography is less accurate for screening of breasts with high mammary gland density [1]. High dense breasts are more often seen in women younger than 50 and are more frequent in Japanese women than in Western women [8]. Furthermore, since the peak age for onset of breast cancer is 40-49 in Japanese women in contrast to the peak age (70-79) in Western women, screening of Japanese women using mammography alone is not expected to be satisfactorily useful [1,2].

Ultrasonography has high accuracy when used for checking breasts with high mammary gland density. Attempts to use ultrasonography as a means of breast cancer screening have already begun to be made. It has been reported that screening with a combination of mammography and ultrasonography has elevated the breast cancer detection rate [9,10,11]. However, neither the specifications of devices for breast ultrasonography nor its procedure and image reading techniques have yet been standardized. Furthermore, the usefulness of ultrasonography as a means of breast cancer screening has not been endorsed.

Nowadays, in both Japan and foreign countries, a prerequisite for performing “cancer screening as a means of countermeasure” under public initiatives is availability of scientific evidence for death rate-reducing effect of the screening [12,13,14]. The Anti-Cancer Basic Act, enforced in April 2007 in Japan, states in Article 18 of Chapter 3

(Basic Policies) that the state and local governments shall promote studies on elucidation of the essential nature of cancer, development of innovative methods for prevention, diagnosis and treatment of cancers, and means for reducing the incidence and death rate of/from cancers and take necessary measures for effective utilization of the outcomes from such studies [15].

In Japan, breast cancer screening was started under the second senile health program in 1987, initially using clinical breast examination. However, since breast cancer screening with clinical breast examination failed to reduce the death rate [16,17], the Guideline on Cancer Screening was partially amended in 2000 to adopt a combination of clinical breast examination and mammography for women aged over 50 (Notification No. 65, Health and Welfare Bureau for the Elderly, MHLW, March 31, 2000). Breast cancer screening for women aged 40-49 previously used clinical breast examination. In 2004, the state partially amended the Guideline on Cancer Prevention-Focusing Health Education and Cancer Screening (Notification No. 0427001, Division of the Health for the Elderly, the Health and Welfare Bureau for the Elderly, MHLW, April 27, 2004) to adopt mammography as a means of screening, as a rule. However, according to the results of randomized comparative studies in Western countries, the death rate-reducing effect of mammography is lower for women aged 40-49 than for women aged over 50 [4,5,6]. Under such circumstances, the interim report from the Cancer Screening Committee, organized under MHLW, made the following two arguments (March 2004) [18].

1. Although ultrasonography is useful in clinical management of breast cancer, no evidence is available for its effect in reducing the death rate from breast cancer.
2. It is desirable from now on to evaluate the usefulness of ultrasonic breast cancer screening and to facilitate standardization of the devices, imaging techniques and image reading techniques related to the screening and to establish a firm diagnostic base for this kind of screening. It is advisable to consider utilization of ultrasonography for screening of individuals with high density breasts whose lesions are difficult to depict by mammography.

Following that report, the report from the research in fiscal 2005 (Chief Researcher: Kiyoshi Kurokawa), carried out within the framework of Research on Strategic Outcomes (Ministry of Health, Labour and Welfare Scientific Research Program), proposed to carry out a randomized comparative study on ultrasonic breast cancer screening [19].

In response to the proposal made by the strategic outcome research group

mentioned above, we have devised a protocol for a study designed to compare the usefulness of ultrasonic breast cancer screening with conventional methods of screening after achieving standardization of the devices, imaging techniques and image evaluation techniques related to ultrasonic screening.

2. Objectives

This study is designed to compare the accuracy and usefulness of breast cancer screening of women aged 40-40 between two groups (a group screened with a combination of mammography and ultrasonography and a group screened with mammography without combined with ultrasonography) after standardization of ultrasonic breast cancer screening procedure.

The primary endpoint of this study is inter-group comparison of the sensitivity, specificity and detection rate. The secondary endpoint is inter-group comparison of the cumulative incidence of advanced* breast cancer during the follow-up period. (*According to the General Rules for Surgical and Pathological Studies on Breast Cancer prepared by the Japanese Breast Cancer Society, breast cancers having metastasized to lymph nodes or distant organs are defined as “advanced breast cancers.”)

The most important indicator of the usefulness of cancer screening is the death rate from a given type of cancer in the population studied. However, in view of the natural history of breast cancer, the four-year period of strategic research is too short to explore a significant inter-group difference in this indicator. Therefore, a system will be established to allow long-term follow-up of the survival/death of the subjects of this study in both groups after completion of this strategic research.

3. Study design

This study is designed to compare the accuracy and usefulness of breast cancer screening of women aged 40-49 between two groups, i.e., the intervention group screened with a combination of mammography and ultrasonography and the non-intervention group screened with mammography. To this end, the below shown two groups are incorporated into this study, with individuals or clusters serving as units.

(1) Ultrasonography combined group (mammography + ultrasonography or mammography + clinical breast examination + ultrasonography)

(2) Ultrasonography uncombined group (mammography alone, or mammography + clinical breast examination)

The targeted number of subjects is 60,000 for each group (120,000 subjects in

total). Of these subjects, 50,000 subjects from each group will receive two sessions of breast cancer screening at an interval of 2 years.

The targeted number of subjects was set on the basis of the forecast as to the capability of elevating the sensitivity (the primary endpoint) by the use of ultrasonography in combination with conventional mammography.

In analysis of the intermediate period cancer (cancer detected before the next session of screening after the latest session of screening yielding a judgment of “abnormality-free”), using the data from the Miyagi Prefecture Regional Cancer Registry, the sensitivity of breast cancer screening with a combination of mammography and clinical breast examination was higher than that of screening with clinical breast examination alone in each age group, but the sensitivity of this combined screening was lower in the age group 40-49 (71.4%) than in any other age group (age group 50-59: 85.8%, age group 60-69: 87.2%) [7]. Of the 20,587 subjects aged 40-49 in that study, 63 had breast cancer during the two-year period after the screening, including 45 cases detected during the screening and 18 cases detected during the intermediate period (sensitivity 45/63=71.4%). Assumed that the addition of ultrasonography to the screening will increase the number of breast cancers detected during screening and that the number of intermediate cancer cases will be halved (from 18 to 9 cases), the sensitivity of the ultrasonography-combined screening will be 86% (54/63). So that this increase in sensitivity can be detected as a significant difference at a significance level of 5% (both-sided) and with a detective power of 80%, the number of breast cancer cases required will be 130. The minimum necessary number of subjects screened is thus estimated to be 42,500 for each group. Therefore, if 50,000 subjects can be enrolled to each group, a valid statistical analysis of the increase in sensitivity following the addition of ultrasonography will be possible.

Then, we analyzed the number of subjects needed for valid analysis of the secondary endpoint (decrease in the cumulative incidence of advanced breast cancer). In the above-mentioned mammography screening group, advanced breast cancer was detected in 12 subjects (27.7%) during the screening and 6 subjects (30.6%) during the intermediate period, with the incidence of advanced breast cancer being 87/100,000 population (18/20587). Assumed that the percentage of advanced breast cancer is the same also in the ultrasonography-combined screening group, the incidence of advanced cancer group in the ultrasonography-combined screening group will be 74/100,000 population (15/20587). So that this difference can be detected as a statistically significant difference at a significance level of 5% (both-sided) and with a detective power of 80%, the number of necessary subjects screened is 760,000 for each group.

Assumed that the survival rate is 96%, 85%, 92% and 50% for patients with early breast cancer detected during screening, advanced breast cancer detected during screening, early breast cancer detected during the intermediate period and advanced breast cancer detected during the intermediate period, respectively, the death rate from breast cancer will be 34/100,000 population for the mammography group and 26/100,000 population for the ultrasonography-combined group. To detect this difference as a statistically significant difference at a significance level of 5% (both-sided) at a detective power of 80%, 760,000 subjects are at least needed for each group. Taken together, these results indicate that a study whose endpoint is the incidence of advanced breast cancer or the death rate from breast cancer after a single session of screening is of little statistical value.

Meanwhile, assumed that the percentage of advanced cancers among all intermediate period cancer cases can be reduced to 10%, the death rate from breast cancer will be 34/100,000 population in the mammography group and 22/100,000 population in the ultrasonography-combined group (36% smaller in the latter group), indicating that the final outcome of this study (reducing the death rate from breast cancer by 30%), a target proposed by the Kurokawa Group, can be achieved. Assumed that screening is continued for 10 years at intervals of 2 years, the cumulative death rate from breast cancer will be 170/100,000 population and 110/100,000 population for the two groups, respectively. To detect this difference as a statistically significant difference at a significance level of 5% (both-sided) and with a detective power of 80%, 55,000 subjects are at least needed for each group. Therefore, if screening can be continued for 10 years, a statistically valid analysis of a decrease in death rate from breast cancer following the addition of ultrasonography will be possible by enrollment of at least 60,000 subjects to each group.

Study designs available for obtaining scientific evidence include individual randomized study, cluster randomized study, cluster non-randomized comparison, etc. From the viewpoint of conducing a high-quality study, validity seems to be highest for the individual randomized study design by which individual subjects, having given informed consent, are assigned at random to one of the ultrasonography-combined group and the ultrasonography-uncombined group. A study design with the second highest validity will be cluster randomized study design by which assignment to the ultrasonography-combined group or the ultrasonography-uncombined group is made at random in units of local community (town or village) or workshop (place of work). If randomized assignment is difficult, cluster non-randomized comparison (a study design by which comparison with the non-intervention group is made, although assignment is

not made) has to be selected.

Another possible study design will be an independent study in which only one type of screening is made (either ultrasonography-combined screening or ultrasonography-uncombined screening), but this study cannot be viewed as a comparative study. For the present study, it is desirable to adopt a study design capable of providing scientific evidence as far as possible. For this reason, participation in this study will be accepted with emphasis laid on the organizations or facilities which intend to perform the study by means of individual randomized comparison, cluster randomized comparison or non-randomized comparison (greatest emphasis laid on the first study design and progressively less emphasis on the next two designs).

4. Criteria for selection of subjects

4.1. Inclusion criteria

(1) Women aged 40-49 at the time of enrollment

(2) Having given informed consent to the study (refer to 13.2 Explanation and consent, Attachment 1, 2, 3, 4 and 5)

4.2. Exclusion criteria

(1) Patients with a history of breast carcinoma (including carcinoma in situ)

(2) Patients having a history of malignancy other than breast cancer during the past 5 years (including carcinoma in situ)

(3) Patients with severe systemic disease unlikely to survival for 5 years or more

4.3. Population for subject recruitment

(1) As a rule, participation in the study is invited from individuals receiving breast cancer screening at the facilities involved in the study.

(2) No restriction is placed on the history of breast cancer screening. However, for subject having a history of breast cancer screening, the year and method of the latest screening are entered in the form.

5. Organization and facilities participating in the study

Participation in the study is invited from organizations and facilities which intend to provide screening using a combination of conventional methods (mammography or mammography + clinical breast examination) and ultrasonography and screening without ultrasonography.

Each participating organization/facility must satisfy the following requirements:

(1) Agrees to the objectives of this study and can cooperate with the study in accordance with the protocol;

(2) Plans to provide breast cancer screening to about 4,000 or more women aged 40-49 during coming two years;

(3) Prepared to perform mammography in accordance with the "Guide to Breast Cancer Screening by Mammography – A Manual for Precision Control, 3rd revised version" [20];

(4) Prepared to perform ultrasonography in accordance with the Anti-Cancer Strategic Research Program "Comparative Study to Evaluate the Usefulness of Ultrasonography as a Means of Breast Cancer Screening" and the "Guideline for Ultrasonic Breast Cancer Screening" edited by the Japan Association of Breast Cancer Screening and Japan Association of Breast and Thyroid Sonology [21];

(5) Satisfies the checklist for breast cancer screening (Guideline on Cancer Prevention-Focusing Health Education and Cancer Screening, partially amended on March 31, 2006, Notification No. 0331003, Division of the Health for the Elderly, the Health and Welfare Bureau for the Elderly, MHLW, April 2004);

(6) Capable of conducting breast cancer screening using the methods specified in 6. Screening methods and providing the services specified in 10.1. Institutional data center.

(7) Preferably being linked to a highly precise regional cancer registration program.

6. Screening methods

6.1 Ultrasonography-combined screening (intervention group)

As a rule, ultrasonography is performed simultaneously with mammography (simultaneous screening with multiple methods). If screening is performed at a facility, the facility needs to have a screening space equipped with a device for ultrasonography (fitted with a body surface probe) and a device for mammography. In case of mobile screening, a bus installed with a device for ultrasonography and a device for mammography is sent to the screening site. Methods of mammography and ultrasonography are specified below. Evaluation of ultrasound images and mammograms is made separately. The subject rated as Category 3 or higher by at least one of the two methods (ultrasonography and mammography) is rated a requiring detailed examination.

6.1.1. Screening with mammography

Mammography is performed in accordance with the "Guide to Breast Cancer Screening by Mammography – A Manual for Precision Control, 3rd revised version." Each facility providing mammography must satisfy the following requirements:

(1) The device for mammography meets the standards on specifications as well as

the standards on X-ray dose and image quality prepared by the Japan Radiological Society;

(2) As a rule, both breasts are imaged in the mediolateral oblique direction and craniocaudal direction;

(3) Mammograms are evaluated by two readers (including at least one physician with adequate experience) under appropriate environments;

(4) Image interpretation shall be performed by classification into categories used for mammography testing.

6.1.2. Screening with ultrasonography

Ultrasonography is performed in accordance with in accordance with the Anti-Cancer Strategic Research Program “Comparative Study to Evaluate the Usefulness of Ultrasonography as a Means of Breast Cancer Screening” and the “Guideline for Ultrasonic Breast Cancer Screening” edited by the Japan Association of Breast Cancer Screening and Japan Association of Breast and Thyroid Sonology. Each facility providing ultrasonography must satisfy the following requirements:

(1) An ultrasonography fitted with a body surface probe and a recorder, satisfying the image quality standards, is used. The probe used should have a frequency about 10 MHz (also 7.5 MHz acceptable for annular array probe) and a visual field width over 35 mm.

(2) Ultrasonography is performed by qualified physicians, laboratory technologists, clinical radiological technologists or nurses having experienced with breast ultrasonography and completed the breast ultrasonography training program.

(3) Judgment is made by a physician experienced with breast ultrasonography.

(4) Categorization for ultrasonography is used when a judgment is made.

6.2. Ultrasonography uncombined screening (non-intervention group)

6.2.1. Screening mammography

Mammography is performed in accordance with the “Guide to Breast Cancer Screening by Mammography – A Manual for Precision Control, 3rd revised version” (Iji Shinpo Pub. Co.). Each facility providing mammography must satisfy the following requirements:

(1) The device for mammography meets the standards on specifications as well as the standards on X-ray dose and image quality prepared by the Japan Radiological Society;

(2) In principle, mediolateral oblique imaging and craniocaudal imaging shall be performed for both breasts.;

(3) Mammograms are evaluated by two readers (including at least one physician with adequate experience) under appropriate environments;
(4) Categorization for mammography is used when a judgment is made.

6.3. Clinical breast examination

Facilities having provided breast cancer screening with clinical breast examination to women aged 40-49 are permitted to provide such screening using the conventional method. In this case, clinical breast examination must be applied equally to both the ultrasonography combined screening group and the ultrasonography uncombined screening group.

7. Study schedule

First half of 2007

(1) Completion of the protocol (authorization by the management committee and the ethics committee)
(2) Standardization of ultrasonography procedure

Latter half of 2007

First screening: Performed on 15,000 subjects each from the ultrasound-combined group (A1) and the ultrasound-uncombined group (B1)

2008

First screening: Performed on 35,000 subjects each from the ultrasound-combined group (A2) and the ultrasound-uncombined group (B2)

2009

(1) First screening: Performed on 10,000 subjects each from the ultrasound-combined group (A3) and the ultrasound-uncombined group (B3)
(2) Second screening: Performed on 15,000 subjects each from the above-mentioned group A1 and group B1

2010

(1) Second screening: Performed on 35,000 subjects each from the above-mentioned group A2 and group B2.
(2) First screening: Not performed

Table 1. Annual plan: Screening schedule

Individual or cluster		2007	2008	2009	2010
Ultrasound-combined (intervention) group	Group 1	A1 (15,000)	A2 (35,000)	A1 (15,000)	A2 (35,000)
	Group 3	None	None	A3 (10,000)	None
Ultrasound-uncombined (non-intervention) group	Group 2	B1 (15,000)	B2 (35,000)	B1 (15,000)	B2 (35,000)
	Group 4	None	None	B3 (10,000)	None

8. Survey and observation

8.1. At the time of enrollment (refer to Attachment 8)

(1) Name of subject
(2) Date of birth
(3) Address (prefecture, ward/city/town/village, street, house number)
(4) Telephone number
(5) History of malignancy: Present/Absent
If present, specify the site:
(6) Interview (history of breast cancer screening, history of menstruation, history of pregnancy and delivery, breast-feeding, familial history of breast cancer, history of breast surgery, presence/absence and nature of subjective symptoms)
- History of breast cancer screening: Absent/Present
If present, the year of latest screening:
Method of screening (clinical breast examination, mammography, ultrasonography
- History of malignancy: Absent/Present
If present, specify the site:
- Subjective symptoms: Absent/Present
If present, specify the nature (mass, tenderness, others:)
(7) Group assigned: Intervention group /Non-intervention group

8.2. Information related to screening results

(1) Date of screening
(2) Name of the screening facility
(Mammography)

(1) Presence/absence of lesions, features of lesions (mass, calcification, others) and category (1, 2, 3, 4, 5), location of lesions

(2) Separate assessment of right and left sides

(Ultrasonography)

(1) Presence/absence of breast lesions (none, mass-forming lesions, non-mass forming lesions), features of lesions, category rated by the examiner (1, 2, 3, 4, 5), overall category rated by the physician in charge of judging (1, 2, 3, 4, 5), location of lesions

(2) Separate assessment of right and left sides

(Clinical breast examination)

(1) Sites checked (A B C D E) and findings (tumor, induration, tenderness, nipple changes, skin changes, others), category (1, 2, 3, 4, 5)

Note: There are no generalized criteria for clinical breast examination category determination, and for this study, the following criteria were used, based on the category determination criteria used for mammography and ultrasound.

Category 1: No abnormality

Category 2: Clearly benign findings

Category 3: Findings that can basically be regarded as benign, but require extensive testing because the possibility of malignancy cannot be ruled out

Category 4: Suspected malignant findings requiring extensive testing

Category 5: Typical malignant findings

(2) Separate assessment of right and left sides

(Overall judgment)

(1) Overall category (1, 2, 3, 4, 5), instruction (no necessity of detailed test, necessity of detailed test)

Note: There are no generalized criteria for overall category determination, and for this study the following criteria were used, based on the category determination criteria used for mammography and ultrasound.

Category 1: No abnormality (extensive testing not required)

Category 2: Clearly benign findings (extensive testing not required)

Category 3: Findings that can basically be regarded as benign, but require extensive testing because the possibility of malignancy cannot be ruled out (extensive testing required)

Category 4: Suspected malignant findings requiring extensive testing (extensive testing required)

Category 5: Typical malignant findings (extensive testing and treatment required)

(2) Separate assessment of right and left sides

8.3. Information related to the individuals receiving a detailed test

(See Attachments 7, 8 and 9)

1. Date of test

2. Testing facility

3. Methods and results of the detailed test

(1) Mammography

(2) Breast ultrasonography

(3) Needle aspiration cytology

(4) Mammary ductography

(5) Mammary ductoscopy

(6) Core needle biopsy

(7) Mammotome biopsy

(8) Surgical biopsy

4. Final diagnosis

(1) Right breast (normal, cancer, fibroadenoma, cyst, mastopathy, others)

(2) Right breast (normal, cancer, fibroadenoma, cyst, mastopathy, others)

5. Outcome

(1) No abnormality (no follow-up until the next screening)

(2) Follow-up

(3) Referral

(4) Treatment

6. If the final diagnosis is breast cancer

(1) Location of cancer

(2) TNM classification

(3) Date and procedure of surgery

(4) Histological diagnosis

(5) Non-surgical treatment provided

8.4. Screening results report to subjects

The screening results are reported to subjects from the screening facility concerned using the form attached (refer to Attachment 10, 11).

8.5. Follow-up

(1) Interview to subjects: Individuals receiving the breast cancer screening from the

latter half of 2007 to 2008 are advised to receive screening again two years later. At the time of the second screening, the presence/absence of breast cancer detected after the first screening is checked.

(2) Survey of inhabitant register: A survey is conducted as to dislocation (from a given local community), death and date of death of individual subjects.

(3) Use of population statistic data for purposes not initially planned: As to the subjects confirmed to be dead by a survey of inhabitant register, an application is filed with the Ministry of Health, Labour and Welfare (MHLW) and the Ministry of Internal Affairs and Communications to obtain approval of the use of the population statistic data for purposes other than the initially planned purposes. If approved, the death slip or tape is accessed to identify the cause of death.

(4) Comparison with the regional cancer register data: An application is filed with the regional cancer registry organization located in the same district as the screening facility to investigate the presence/absence (and the date of onset, if any) of breast cancer among the subjects of this study.

9. Follow-up method

Follow-up is made using two methods, i.e., direct contact (interview and mail) with the subject (or her relative) and comparison with external data.

When communicating directly with subjects (or persons involved), at the point at which 20 months have passed since they underwent the first breast cancer screening, a sealed letter shall be sent to the subjects to encourage them to undergo a second breast cancer screening. During the second screening, the presence/absence of breast cancer diagnosed after the first screening is checked. For subjects who do not receive the second screening, the presence/absence of breast cancer diagnosed after the first screening is investigated over telephone.

Comparison with external data means checking for move-outs or death of subjects by means of access to the inhabitant register, access to the death slip to confirm death (and its cause) and access to regional cancer register to check for beast cancer among the subjects. The step to obtain approval of each comparison to external data is taken by the Central Data Center, and comparison is practiced after approval is obtained (refer to “8.5. Follow-up” for details).

10. Safety management

The Anti-cancer Strategic Research Ethics Committee (hereinafter called “the Ethics Committee”) holds its meetings in accordance with the Rules for the Ethics

Committee organized within the Japan Cancer Society (hereinafter called “the Society”) pursuant to the Manual for Anti-cancer Strategic Research within the framework of the Third Comprehensive Anti-Cancer Strategic Research Program, Ministry of Health, Labour and Welfare (Notification No. 1012001, Health Policy Bureau, MHLW, October 12, 2006). The Strategic Research Leader (hereinafter called “the Research Leader”) submits the study protocol and periodically reports the study status, adverse events, etc. to the Ethics Committee. The Ethics Committee checks and approves the protocol and advises the Research Leader to modify or discontinue the study.

10.1. Duties of the responsible person of each participating organization (breast cancer screening facility)

The person responsible at the participating organization (the breast cancer screening facility) establishes a system for close cooperation with the Central Data Center. If any inquiry about the study is received from a subject, the responsible person takes necessary and appropriate actions. If a problem related to safety management has been identified following onset of a serious advent possibly affecting continuation of the study, the responsible person takes necessary and appropriate actions and ensures safety of subjects. At the same time, the responsible person reports such an event to the Research Leader promptly.

The intervention made during this study is ultrasonic breast cancer screening and it is carried out within the scope of routine clinical test practice. For this reason, no serious adverse event is expected to arise from this study. However, if a serious adverse event has arisen during the breast cancer screening, the person in charge of breast cancer screening in the screening facility concerned takes necessary and appropriate actions and ensures the safety of the subjects. Furthermore, the person in charge of breast cancer screening submits a report on such a event to the responsible person of a given organization, who then reports the event to the Research Leader without delay.

10.2. Duties of Research Leader

Upon onset of a serious adverse event or an event which can affect continuation of the study, the Research Leader collects information about the event and gives necessary instructions to co-researchers in charge. At the same time, the Research Leader reports the event to the Data Center and other participating organizations, so that the information pertaining to the event can be shared. The event is also reported to the Society, the Management Committee and the Ethics Committee.

11. Statistical analyses

11.1. Major statistical analyses

Primary endpoints (sensitivity, specificity and detection rate) are compared between the intervention group and the non-intervention group. As a secondary endpoint, the cumulative incidence of advanced breast cancer during the follow-up period is compared. Through these statistical analyses, the accuracy and usefulness of ultrasonic breast cancer screening are evaluated. The Central Data Center conducts the above-mentioned statistical analyses according to the statistical analysis protocol and reports the results to the Secretariat of the Study Group. The statistical analysis protocol is prepared by the Statistical Analysis Manager with the cooperation of experts in biological statistics.

11.2. Follow-up

The sensitivity and specificity of breast cancer screening can be analyzed on the basis of the first screening results and the results from the survey of intermediate period cancer during the second screening. However, the most important indicator of the usefulness of cancer screening is the death rate from a given type of cancer in the population studied. To compare the death rate from a given type of cancer between the intervention group and the non-intervention group, comparison of data with the regional cancer registry data, etc. is necessary. For this reason, the Central Data Center shall carry out the application procedures for cross-checking with resident registries and issuing record deletion certificates, use of Vital Statistics, and cross-checking with local cancer registry data, view these data, and carry out cross-checking.

In view of the natural history of breast cancer, the four-year period of strategic research is too short to explore a significant inter-group difference in this indicator. Therefore, a system will be established to allow long-term follow-up of the survival/death of the subjects of this study in both groups after completion of this strategic research.

12. Study period

July 1, 2007 through March 31, 2013

13. Ethical consideration: Informed consent

All researchers involved in this study follow the Declaration of Helsinki when pursuing the study. They observe the Ethical Guideline on Clinical Studies (Ministry of Education, Culture, Sports Science and Technology and Ministry of Health, Labour and

Welfare) and ensure the ethical acceptability and safety of the study and the scientific validity and reliability of the outcome.

13.1. Use of individual information

The data collected for this study are managed in accordance with the data handling manual. This study follows the ethical guideline on clinical studies and abides by its provisions related to protection of individual information. The Research Leader is responsible for safe management of individual information arising from the study.

13.2. Explanation and consent

Explanation about the study to obtain informed consent is given to each subject orally and in writing, as a rule. Informed consent in writing from the subject is indispensable.

The following are major pieces of information to be supplied to the subject (refer to Appendix 1, 2, 3, 4 and 5).

(1) Objectives, significance and outline of the study;
(2) Item and method of investigation: Screening results and detailed tests results for those rated as requiring detailed examination:
(3) Follow-up survey: Onset of breast cancer and survival after the screening;
(4) That the subject can participate in the study at her own discretion;
(5) That failure to consent to the study does not cause any disadvantageous treatment to the subject;
(6) That the consent to the study once issued can be canceled any time by the subject or her proxy consenter without suffering any disadvantage;
(7) The reason for selection of the subject for this study;
(8) Name and title of researchers and others;
(9) Anticipated outcome of this study, benefits expected from participation in this study, possible risks and inevitable uncomfortable conditions, and measures taken after the end of the study;
(10) That the subject and her proxy consenter or the like can obtain or access to the information related to the plan and methods of this study within a range causing no problem in protection of the individual information of other subjects or in preservation of the novelty of this clinical study;
(11) That the individual information arising from this study is managed strictly and used only for this study;
(12) That the results of the study may be published after measures are taken

to avoid identification of individual subjects;

(13) Presence/absence of compensation related to the study;

(14) Information about where the subject should contact for inquiry, reporting claims, etc.

13.3. Authorization of the protocol

The plan of this study needs to be inspected and approved by the Ethics Committee.

13.4. Compensation related to the study

If the subject has sustained health hazards directly associated with this study, no special compensation will be made. The person in charge will deal with such hazards within the framework of healthcare under health insurance.

14. Discontinuation of the study

If the Ethics Committee advises the Research Leader to discontinue the study in accordance with the Rules of the Ethics Committee set forth by the Society, the Research Leader immediately considers the necessity of discontinuation on the basis of the results of discussions at the Ethics Committee. If discontinuation is deemed to be valid, the Research Leader decides discontinuation of the study.

15. Storage, disposal or deletion of records

For central management of electronic data of registered subjects, the Data Center manages the data according to the Data Handling Manual. The Data Handling Manual is prepared by the Data Center. Access to the data is permitted only to those certified with ID and password. Security measures (e.g., firewall installment) are taken to avoid illegal access to the data. The Data Center, the Study Group Secretariat and the Facility Manager of the participating organization continue to store the dataset or information prepared for this study after completion of the study. All participants are required to observe the Individual Information Protection Act and stores the datasets, information, etc. in accordance with the methods stipulated in the Data Storage Manual. The duration of storage of research datasets, information, etc. is specified in the Data Storage Manual.

16. Data management and monitoring

To facilitate smooth research, an institutional data center is organized in each

participating organization. In addition, the Central Data Center is established. Each data center functions as shown below (refer to Attachment 12, 13).

16.1. Institutional data center

(1) Invites subjects to this study (white advising people to receive breast cancer screening);

(2) Checks the eligibility of candidate subjects at the screening site and explains the objectives of this study to eligible individuals, followed by collection of informed consent to the study in writing;

(3) Conducts screening with the assigned method and reports the results to participants;

(4) Advises subjects rated as requiring detailed examination to receive the examination and checks the detailed test results;

(5) Stores documents (consent form, screening results, detailed test results);

(6) Enter data of subjects (information for subject identification, background information, screening results and detailed test results) and send them to the Central Data Center;

(7) Receives a consent cancellation form and stores it, followed by reporting to the Central Data Center;

(8) Conducts precision control of screening. To this end, periodically checks the maintenance/inspection history of devices, methods of test, etc., referring to the study protocol and the manual, and reports the results of check to the Central Data Center periodically (four times a year). Cooperate with sampling test for evaluation of mammograms and ultrasound images;

(9) Advises the subjects receiving the first breast cancer screening in latter half of 2007 to 2008 (Group A1, A2, B1 and B2 mentioned below) to receive the second screening 2 years after the first screening. At that time, the presence/absence of breast cancer detected after the first screening is checked (refer to “8.5. Follow-up” and “9. Follow-up methods” for details).

16.2. Central Data Center

(1) Prepares documents (leaflet on the study, request to participation in the study, consent form, etc.) to be delivered to participants, prints them, and send them to each screening organization.

(2) Organizes training sessions for advisers (who explain the study to subjects) at each institutional data center;

(3) Organizes the “Call Center” to deal with inquiry from participants (and candidates)

about the study and consent;

(4) Prepares the randomization table and the assignment instruction form separately in accordance with the manual for use at facilities adopting the individual randomization design. The randomization table lists the steps to be taken for assignment to all participants at a given facility. This table is stored at the Central Data Center. The Central Data Center prepares the assignment instruction form and dispatches it to the institutional data centers. The assignment instruction form instructs either “mammography + ultrasonography” or “mammography alone”. Participants receive screening in accordance with this instruction. The assignment instruction form is sealed into an envelope for each subject, and the envelope carries a serial number.

(5) Performs randomized assignment to the organizations adopting cluster randomization design in according with the manual prepared for each cluster, and reports it to the institutional data center.

(6) Designs and develops a database for data compilation and processing after receipt of data from institutional data centers. Controls the database thereafter.

(7) Checks the acceptability of the data received from institutional data centers, and makes inquiry to the institutional data center, data correction and/or data editing as needed.

(8) Follows the individual information management rules at a given facility and the Standard Operating Procedure (SIP) for the entire study. Adopts a standard encrypt system, adopted by enterprises, governmental organs, etc. worldwide, for telecommunication (refer to Attachment 14);

(9) Processes the above-mentioned data periodically and reports the results to the Research Leader and the institutional data centers;

(10) Sends the Data Manager to institutional data centers for data quality control and assistance. Practice precision control of screening by processing and analyzing the reports from institutional data centers pertaining to maintenance/inspection of devices, screening procedure, etc. and conducts on-site audit (approximately once a year). Submits reports on these actions to the Research Leader and the Precision Control and Safety Evaluation Committee;

(11) When contracted by a participating organization to act as a representative institution, communicate directly by mail or telephone with individuals undergoing screening

(12) Takes steps related to access to inhabitant register data, use of population statistical data for purposes other than initially planned purposes and comparison to regional cancer registry data (submission of application for approval, access to data, and

comparison of these data with the data from screening subjects);

(13) Conducts statistical analysis of data from this study using the methods set forth in "11. Statistical analyses" and reports the results to the Research Leader.

The Central Data Center is nominated after open invitation in accordance with the rules set forth by the Society.

17. Review and modification of the protocol

If the finding from periodical monitoring or the onset of serious adverse events indicates a problem or question in continuation of the study, the Ethics Committee immediately advises the Research Leader to consider modification of the protocol in accordance with the rules of the Ethics Committee prepared by the Society. The Research Leader immediately considers modification of the protocol on the basis of the results of discussions at the Ethics Committee. If modification of the protocol is deemed as acceptable, the Research Leader drafts a modified protocol and submits it to the Ethics Committee. The Ethics Committee inspects it. If it is approved by the Ethics Committee, the modification is adopted after discussions at the Management Committee.

18. Additional compound study

The Research Leader and the co-researchers may plan additional compound studies if deemed necessary. After securing coherence with the present study, a plan for each additional study is prepared.

The primary analysis in the original report revealed that the sensitivity was significantly higher in the intervention group than in the control group. However, results according to breast density were not described in detail. Study addressing issues related to performance of each modality according to differences in breast density was approved on January 18, 2019.

19. Publication of study outcomes

Major outcomes of this study will be contributed to peer-reviewed English journals after completion of final analysis. Publication of the results about primary endpoints (sensitivity, specificity and detection rate) and the secondary endpoint (cumulative incidence of advanced breast cancer during the follow-up period) will be possible within several years after completion of this study.

Although the primary objective of this study is to evaluate the usefulness of ultrasonic breast cancer screening, it is impossible to collect data showing death

rate-reducing effect of screening within the 4-year period of this study, in view of the natural history of breast cancer. For this reason, a system for long-term follow-up of subjects from both groups as to survival/death after the end of the study will be established, and the data collected from such follow-up will be analyzed and reported.

Although the results about standardization of ultrasonic breast cancer screening procedure are not major outcomes from this study, they will be useful in improving the accuracy of breast cancer screening in Japan. For this reason, co-researchers will be able to publish these secondary outcomes and the processes of such an aspect of this study in professional medical journals, meetings of professional organizations or the if the Research Leader permits it.

The authorship of each academic paper related to this study will be decided separately through negotiations among the MHLW (Cancer Countermeasures Promotion Office, General Affairs Division, Health Policy Bureau), the Society and the Study Group Secretariat. The co-authors of a paper are confined to those who checked the manuscript before contribution and agreed to it. No one other than the Research Leader may directly receive the results of data processing or analysis from the Data Center without the consent of the Study Group Secretariat and the Research Leader.

20. Study participants other than subjects and the study organization

20.1. Study participants

Participants in this study include physicians specializing in breast cancer, nurses, clinical radiological technologists, laboratory technologists, epidemiologists, biological statisticians, public officials in charge of regional health, staff of the Study Group Secretariat, staff of the participating organizations, data center staff, and others.

20.2. Study organization and roles

(1) Study organization outlined

The organization for this study consists of the Research Leader, sub-investigators, participating organizations (breast cancer screening facilities), Study Group Secretariat, Study Group Management Committee, Data Monitoring Committee, Educational Program Committee, Precision Control and Safety Evaluation Committee and Statistical Analysis Committee (Table 2). In accordance with the Manual for Strategic Research on Countermeasures against Cancer within the framework of the Third Comprehensive Anti-Cancer Strategic Research Program, Ministry of Health, Labour and Welfare, the following committees have been organized: Management Committee, Ethics Committee, Evaluation Committee and Progress Control Committee. This study group will implement the protocol under supervision by these committees.

(2) Strategic Research Leader

Noriaki Ohuchi, Professor, Department of Surgical Oncology, Tohoku University Graduate School of Medicine

Strategic Research Leader has the following duties:

- Organizes the study group and implement the study in accordance with this protocol;
- Organizes the Study Group Secretariat for clerical work, e.g., dealing with inquiry about the protocol, adverse events, modification of the protocol, etc.
- Nominates a biological statistician for the study group to devise statistical analysis plan and implement final statistical analyses.

(3) Sub-investigators

So that this study can proceed smoothly, the Research Leader nominates sub-investigators. Sub-investigators serve as members of one of the following research groups and contribute to implementation of the study plan:

1. Research group related to monitoring: The Data Monitoring Committee is organized to perform periodical data management and monitoring for this study;
2. Research group related to educational programs: The Educational Program Committee is organized to facilitate standardization of ultrasonic screening procedure and establishment of an education and training system;
3. Research group related to precision control and safety evaluation: The Precision Control and Safety Evaluation Committee is organized to evaluate the precision and safety of screening by the participating organizations (breast cancer screening facilities);
4. Research group related to statistical analyses: The Statistical Analysis Committee is organized to devise and discuss the plans on statistical analyses of data collected during this study and on follow-up surveys.

(4) Participating organizations (breast cancer screening facilities)

- Serve as managers for study implementation;
- Invite women aged 40-49, gathering at breast cancer screening sites, to participate in this study;
- Conduct mammography and ultrasonography on women who have given informed consent, fill in the results in the screening record report form (prepared by the Secretariat for this study) and send the report to the Central Data Center.
- Advise subjects, rated as requiring detailed examination, to receive the examination and check and report the results to the Central Data Center.

- Advise the subjects receiving breast cancer screening in 2007 or 2000 to receive the second screening 2 years later, and check the presence/absence of breast cancer detected during this period.
- Participating organizations can request the Central Data Center to act as their representative in conducting surveys of the above participants.

(5) Study Group Secretariat

- Take overall steps needed for implementation of this study, under direction of the Research Leader, and organize and manage the Study Group Management Committee. Also deal with inquiry, etc. from participating organizations, as needed. The Research Leader supervises the participating organization so that the study can be implemented and completed appropriately. Upon appearance of an event or case not defined in the protocol, the Research Leader determines an urgent action if needed and takes such an action. If the Research Leader cannot fulfill the duties, the Assistant Strategic Research Leader acts as the Research Leader.

(6) Study Group Management Committee

The Study Group Management Committee consists of sub-investigators and managers for study implementation at participating organizations. This committee holds meeting in response to request by the Research Leader to prepare and modify the protocol, manage the study group (including discussions about the reports from data centers) and discuss matters related to study implementation.

- The chairman of this committee is nominated by the Research Leader.
- The chairman convenes meetings of this committee and submits the minutes immediately after completion of a meeting to the Research Leader.

(7) Data Monitoring Committee

The Data Monitoring Committee monitors the study.

- This committee consists of the Research Leader and sub-investigators.
- The committee is operated not only in the form of meetings but also by discussions over telephone, mail, E-mail, etc.
- The progress of the study is checked by inviting reports by the Central Data Center and monitor staff as to data collection, satisfaction of the inclusion criteria and data on serious adverse events, with a goal of confirming that the study is being implemented in a safe and appropriate manner. If a problem affecting the scientific or ethical aspects of the study and casting doubt on continuation of the study has been identified, how to resolve the issue is discussed immediately with the Central Data Center, etc.

- The chairman of this committee is nominated by the Research Leader.
- The chairman convenes meetings of this committee and submits the minutes immediately after completion of a meeting to the Research Leader.

(8) Educational Program Committee

This committee is organized under the direction of the Research Leader. It facilitates standardization of ultrasonic breast cancer screening during this study and organizes training for physicians and technologists. The committee tests the validity and feasibility of training programs by trying them at organizations to which the researchers belong or are affiliated, so that the improved versions of the programs may be later disseminated across the country. At the time of breast cancer screening conducted by the participating organizations, the screening staff (physicians or technologists) are required to have completed the training course provided by the Educational Program Committee. As set out in the item in “6. Screening Methods,

- The chairman of this committee is nominated by the Research Leader.
- The chairman convenes meetings of this committee and submits the minutes immediately after completion of a meeting to the Research Leader.

(9) Precision Control and Safety Evaluation Committee

The Precision Control and Safety Evaluation Committee evaluates the precision of screening conducted by participating organizations (breast cancer screening facilities). As needed, the committee makes on-site inspection of the facilities. If evaluation of data collected as to adverse events is requested by the Research Leader, this committee discusses measures to be taken for the events and analyzes the causal relationship to this study, followed by reporting of the results to the Research Leader.

- The chairman of this committee is nominated by the Research Leader.
- The chairman convenes meetings of this committee and submits the minutes immediately after completion of a meeting to the Research Leader.

(10) Statistical Analysis Committee

- The Statistical Analysis Committee is organized to devise and discuss the plans on statistical analyses of data collected during this study and on follow-up surveys. The committee analyzes the precision, usefulness, etc. of breast cancer screening using the data stored and managed at the Central Data Center and reports the results to the Research Leader.
- The chairman of this committee is nominated by the Research Leader.
- The chairman convenes meetings of this committee and submits the minutes immediately after completion of a meeting to the Research Leader.

21. References

1. Oshima A, Kuroishi T, Tajima K. *Cancer incidence mortality and survival, Cancer statistics in Japan*. Tokyo, Japan: Shinohara-Shinsha Publishing Inc; 2004.
2. Tajima K Hirose K. Trends in breast cancer incidence and mortality in Japan. *Japanese Journal of Clinical Medicine.* 65 (Suppl 6): 15-21, 2007(in Japanese).
3. Minami Y, Kakugawa Y, Ohuchi N. Lifestyle factors responsible for the increasing incidence of breast cancer. *Japanese Journal of Clinical Medicine.* 65 (Suppl 6): 213-219, 2007 (in Japanese).
4. Smart CR, Hendrick RE, Rutledge JH, 3rd, Smith RA. Benefit of mammography screening in women ages 40 to 49 years. Current evidence from randomized controlled trials. *Cancer.* 75:1619-1626, 1995.
5. Hendrick RE, Smith RA, Rutledge JH, 3rd, Smart CR. Benefit of screening mammography in women aged 40-49: a new meta-analysis of randomized controlled trials. *J Natl Cancer Inst Monogr.* 22: 87-92, 1997.
6. Moss SM, Cukcle H, Johns L, Waller M, Bobrow L. Effect of mammographic screening from age 40 years on breast cancer mortality at 10 years' follow-up: a randomised controlled trial. *Lancet.* 368: 2053-2060, 2006.
7. Ohuchi N, et al. Research on improving the accuracy, effectiveness and efficiency in breast cancer screening. Annual report of the Second Term Comprehensive Control Research for Cancer Health and Labour Sciences Research Grant, 2007 (in Japanese).
8. Hasegawa S, Ohnuki K, Nagakubo J, et al. Evaluation of breast cancer visualization ability of mammography by age group and by breast constitution (mammary gland/fat ratio). J Jpn Assoc Breast Cancer Screen. 12:101-107, 2003 (in Japanese).
9. Takebe K, Nakamura K, Misao K. Usefulness of whole-breast scanning by breast cancer screening of young women. *J Jpn Assoc Breast Cancer Screen.* 9:155-160, 2000 (in Japanese).
10. Tsuchiya J, Asano M, Tachibana S, et al. Usefulness of ultrasound mass screening for breast cancer in women aged under 50. *J Jpn Assoc Breast Cancer Screen.* 10:185-193, 2001 (in Japanese).
11. Yamasaki M, Nasu S, Koga S, et al. Detection Rate of Breast Cancer by Mammography and Ultrasonography Screening in Women Aged Between 40 and 49. *J Jpn Assoc Breast Cancer Screen.* 11:265-269, 2002 (in Japanese).
12. WHO. National Cancer Control Programmes: Policies and managerial guidelines. 2nd Ed. 2002. (http://www.who.int/cancer/nccp/en/). Accessed June 14, 2007.
13. Hisamichi S. Efficacy evaluation of the new cancer screening method. Report by

the research projects on cancer screening rationalization. Japan Public Health Association, 2001 (in Japanese).

14. Sobue T. Develop the appropriate method for implementing and evaluating cancer screening. Annual report of the Second Term Comprehensive Control Research for Cancer Health and Labour Sciences Research Grant, 2004 (in Japanese).
15. Cancer Control Act, https://www.mhlw.go.jp/english/wp/wp-hw4/dl/health_and_medical_services/P73.pdf.Accessed June, 2007.
16. Ota J, Horino T, Taguchi T, Ishida T, et al. Mass screening for breast cancer: Comparison of the clinical stages and prognosis of breast cancer detected by mass screening and in out-patient clinics. *Jpn J Cancer Res.* 80: 1028-1034, 1989.
17. Kanemura S, Tsuji I, Ohuchi N, Takei H, Yokoe T, Koibuchi Y, Ohnuki K, Fukao A, Satomi S, Hisamichi S. A case control study on the effectiveness of breast cancer screening by clinical breast examination in Japan. *Jpn J Cancer Res.* 90: 607-613, 1999.
18. An interim report by a Health, Labuor and Welfare Ministry panel on cancer screening. To review the breast and uterus cancer screening basing Health and Medical Service Act for the Aged, 2004 (in Japanese).
19. Kurokawa K. The Third Term Comprehensive Control Research for Cancer from the Ministry of Health, Labour and Welfare of Japan, 2006.
20. Ohuchi N. eds. *Manual of quality control. Guide to Breast Cancer Screening by Mammography – A Manual for Precision Control.* 3rd ed revised version. Tokyo, Japan: Japan Medical Journal; 2007 (in Japanese).
21. The Japan Association of Breast Cancer Screening and Japan Association of Breast and Thyroid Sonology. Comparative Study to Evaluate the Usefulness of Ultrasonography as a Means of Breast Cancer Screening” and the Guideline for Ultrasonic Breast Cancer Screening, Anti-Cancer Strategic Research Program, 2006 (in Japanese).

Appendices: Summary of protocol changes

(1) January 14, 2010; Amendments to Study Protocol		
Item<br>(All pages and paragraph are in Japanese version)	Before amendment<br>(underlined portion amended)	After amendment (underlined portion amended)
Cover page	Ver. 1.0<br>June 14, 2007	Ver 2.0<br>January 14, 2010
5. Organization and facilities participating in the study	(6) Capable of conducting breast cancer screening using the methods specified in 6. Screening methods and providing the services specified in 16.1. Institutional data center.	(6) Capable of conducting breast cancer screening using the methods specified in 6. Screening methods and providing the services specified in 10.1. Institutional data center.
6.1.1 Screening with mammography	(4) Image interpretation shall be performed by classification into categories.	(4) Image interpretation shall be performed by classification into categories used for mammography testing.
6.2.1 Screening mammography	(2) In principle, mediolateral oblique imaging and craniocaudal imaging shall be performed for both breasts.	(2) In principle, mediolateral oblique imaging and craniocaudal imaging shall be performed for both breasts.<br>[The minor changes in the Japanese, but the English is not changed]
8.2 Information related to screening results (Clinical breast examination)	(An annotation has been added to (1))	Note: There are no generalized criteria for clinical breast examination category determination, and for this study, the following criteria were used, based on the category determination criteria used for mammography and ultrasound.<br>Category 1: No abnormality<br>Category 2: Clearly benign findings<br>Category 3: Findings that can basically be regarded as benign, but require extensive testing because the possibility of malignancy cannot be ruled out<br>Category 4: Suspected malignant findings

		requiring extensive testing<br>Category 5: Typical malignant findings
8.2 Information related to screening results (Overall judgment)	(An annotation has been added to (1))	Note: There are no generalized criteria for overall category determination, and for this study the following criteria were used, based on the category determination criteria used for mammography and ultrasound.<br>Category 1: No abnormality (extensive testing not required)<br>Category 2: Clearly benign findings (extensive testing not required)<br>Category 3: Findings that can basically be regarded as benign, but require extensive testing because the possibility of malignancy cannot be ruled out (extensive testing required)<br>Category 4: Suspected malignant findings requiring extensive testing (extensive testing required)<br>Category 5: Typical malignant findings (extensive testing and treatment required)
9. Follow-up method, second paragraph	When communicating directly with subjects (or persons involved), at the point at which 20 months have passed since they underwent the first breast cancer screening, a sealed letter shall be sent to the subjects to encourage them to undergo a second breast cancer screening.	When communicating directly with subjects (or persons involved), at the point at which 20 months have passed since they underwent the first breast cancer screening (comma deleted in the Japanese protocol) a sealed letter shall be sent to the subjects concerned encouraging them to undergo a second breast cancer screening.
9. Follow-up method, third paragraph	Comparison with external data means checking for changes of address or death of subjects by means of access to	Comparison with external data means checking for move-outs or death of subjects by means of access to the inhabitant register,

	the inhabitant register,	
20.2 Study organization and roles (8) of the Educational Program Committee	At the time of breast cancer screening conducted by the participating organizations, the screening staff (physicians or technologists) are required to have completed the training course provided by the Educational Program Committee.	At the time of breast cancer screening conducted by the participating organizations, the screening staff (physicians or technologists) are required to have completed the training course provided by the Educational Program Committee. As set out in the item in "6. Screening Methods,"
20.2 Study organization and roles (9) of the Quality Control and Safety Evaluation Committee	If evaluation of data collected as to adverse events is requested by the principal investigator, this committee discusses measures to be taken for the events and analyzes the causal relationship to this study, followed by reporting of the results to the principal investigator.	If evaluation of data collected as to adverse events is requested by the Research Leader, this committee discusses measures to be taken for the events and analyzes the causal relationship to this study, followed by reporting of the results to the Research Leader.
"Table 2. Study Organization"		Changes in the names of institutions with which researchers are affiliated, changes in staff members responsible due to transfers, updating of changes in positions
(2) July 29, 2010; Amendments to name list		
Cover page<br>Data and version	Ver. 2.0<br>January 14, 2010	Ver. 3.0<br>July 29, 2010
"Table 2. Study Organization"		Changes in the names of institutions with which researchers are affiliated, changes in staff members responsible due to transfers, updating of changes in positions
(3) September 15, 2010		
16.2 Central Data Center	(11) Takes steps related to access to inhabitant register data, use of population	(11) When contracted by a participating organization to act as a representative institution, communicate directly by mail

	statistical data for purposes other than initially planned purposes and comparison to regional cancer registry data (submission of application for approval, access to data, and comparison of these data with the data from screening subjects);<br>(12) Conducts statistical analysis of data from this study using the methods set forth in “11. Statistical analyses” and reports the results to the Research Leader.	or telephone with individuals undergoing screening<br>(12) Takes steps related to access to inhabitant register data, use of population statistical data for purposes other than initially planned purposes and comparison to regional cancer registry data (submission of application for approval, access to data, and comparison of these data with the data from screening subjects);<br>(13) Conducts statistical analysis of data from this study using the methods set forth in “11. Statistical analyses” and reports the results to the Research Leader.
20.2 Study organization and roles	(4) 〜Advise the subjects receiving breast cancer screening in 2007 or 2000 to receive the second screening 2 years later, and check the presence/absence of breast cancer detected during this period.	(4) Advise the subjects receiving breast cancer screening in 2007 or 2000 to receive the second screening 2 years later, and check the presence/absence of breast cancer detected during this period. Participating organizations can request the Central Data Center to act as their representative in conducting surveys of the above participants.
(4) December 16, 2011; Amendments to name list only		
Cover page	Ver. 3.0<br>July 29, 2010	Ver. 4.0<br>December 16, 2011
“Table 2. Study Organization”		Changes in the names of institutions with which researchers are affiliated, changes in staff members responsible due to transfers, updating of changes in positions
(5) July 15, 2012		
Cover page	Ver. 4.0<br>December 16, 2011	Ver. 5.0<br>July 15, 2012
Overview Study	July 2007 to March 2011	July 2007 to March 2013

Period		
11.2 Follow-up	~ For this reason, the Central Data Center shall carry out the application procedures for cross-checking with resident registries and issuing record deletion certificates, use of Vital Statistics for a purpose other than the intended use, and cross-checking with local cancer registry data, view these data, and carry out cross-checking.	~ For this reason, the Central Data Center shall carry out the application procedures for cross-checking with resident registries and issuing record deletion certificates, use of Vital Statistics, and cross-checking with local cancer registry data, view these data, and carry out cross-checking.
“Table 2. Study Organization”		Changes in the names of institutions with which researchers are affiliated, changes in staff members responsible due to transfers, updating of changes in positions
⑹ January 18, 2019; Amendments to the sub-study approved		
Cover page	Ver. 5.0<br>July 15, 2012	Ver. 6.0<br>January 18, 2019
18.Additional compound study	The Research Leader and the co-researchers may plan additional compound studies if deemed necessary. After securing coherence with the present study, a plan for each additional study is prepared.	The Research Leader and the co-researchers may plan additional compound studies if deemed necessary. After securing coherence with the present study, a plan for each additional study is prepared.<br>The primary analysis in the original report revealed that the sensitivity was significantly higher in the intervention group than in the control group. However, results according to breast density were not described in detail. Study addressing issues related to performance of each modality according to differences in breast density was approved on January 18, 2019.

1056

The Health, Labour and Welfare Research Grant

(The Third Comprehensive Anti-Cancer Strategic Research Program)

“A Comparative Study to Evaluate the Usefulness of Ultrasonography as a Means of Breast Cancer Screening”

Japan **S**tra**t**egic **A**nti-Cancer **R**andomized **T**rial

(J-START)

Evaluation of adjunctive Ultrasound for Breast Cancer Detection among Women aged 40-49 with Varying breast Density Undergoing Screening Mammography: A Randomized Controlled Trial
Statistical Analysis Plan (SAP)

Study chair: Noriaki Ohuchi, MD, PhD,

Professor, Department of Breast and Endocrine Surgical Oncology, Tohoku University

December 31, 2013 Version 1

January 27, 2014 Version 1.1

August 20, 2018 Version 2.0

January 18, 2019 Version 3.0

1. Definition of the Statistical Analysis Plan (SAP) ... 3
2. Study Summary ... 3
2.1 Objectives ... 3
2.2 Study design ... 3
2.3 Criteria for Selection of Subjects ... 4
2.3.1 Age ... 4
2.3.2 Inclusion criteria ... 4
2.3.3 Exclusion criteria ... 4
2.4 Endpoints ... 4
2.4.1 Primary endpoints ... 4
2.4.2 Secondary endpoint ... 4
2.5 Sample size ... 4
3. Planned Period of the Study ... 6
3.1 Schedule of the first and second screening and the number of subjects to be enrolled ... 6
3.2 Secondary endpoint ... 6
4. Data Monitoring ... 6
5. Definitions and Data Handling Rules for Analyses ... 7
5.1 Definitions of screening intervals, breast cancer detected at the first screening, and interval breast cancer ... 7
5.2 Rules for determining the presence or absence of breast cancer ... 8
5.2.1 Had Breast Cancer (cancer detected at the first screening, interval cancer) ... 8
5.2.2 Did not have Breast Cancer (not breast cancer) ... 8
5.2.3 Had unknown status (unknown) ... 8
5.3 Data review in a blinded manner (Case ascertainment and validation) ... 9
5.4 TNM stages and histological classification data check ... 10
5.5 Breast density classification ... 10
6. Analysis Methods ... 10
6.1 Statistical analyses of the primary endpoints ... 10
6.2 Pooling data for analyses ... 11
6.3 Analysis set ... 11
6.4 Preparation of SAS data set for analysis (Data Source) ... 11
6.4.1 Verification of compliance with randomization ... 11
6.4.2 Subjects not meeting the eligible criteria ... 12
6.4.3 Withdrawal of consent ... 12
6.4.4 Deviation ... 12
7. Statistical Analyses ... 12
7.1 Comparison of characteristics of baseline demographic variables and he distributions of baseline

factors 12
7.2 Analyses of a detection rate, sensitivity and specificity of the first screening and calculation methods for 95% confidence intervals (CIs) 13
7.2.1 Definitions and calculation formulas 13
7.2.2 Method of the primary analysis taking account of correlations within the cluster 13
8. Statistical Analysis Rules 14
9. Technical Matters 14
10. Validation of Analysis Results 14
11. Changes to Planned Analyses 15
12. List of the Statistical Analysis Committee Members 15
13. Development Group for Analysis Procedures for First Screening Results/ Operation Records 16
14. Breast Density Classification Quality Manager Team 16
15. Summary of changes from version 1.0 to version 3.0 16

1. Definition of the Statistical Analysis Plan (SAP)

To specify the details of primary and secondary endpoints of statistical analyses in the Health Labour Sciences Research Grant (the third-term comprehensive control research for cancer), “a Comparative Study to Evaluate the Efficacy of Ultrasonography in Breast Cancer Screening,” J-START.

This SAP for “The performance of adjunctive ultrasonography in breast cancer screening according to breast density from the Japan Strategic Anti-cancer Randomized Trial (J-START)”, is based on the protocol and describes details on statistics and analyses to evaluate the performance of adjunctive ultrasonography to mammography for breast cancer screening according to difference in breast density and study groups, as well as on each modality.

The statistical analyses method for secondary endpoint are not included in this SAP and will be addressed in separate publications in the future as data become available.

2. Study Summary

This is a comparative study to evaluate the diagnostic accuracy and efficacy of a breast cancer screening method in women aged 40 to 49 years between a group using mammography in combination with ultrasonography (intervention group) and group using mammography alone (non-intervention group). Hence, the following two groups will be established individually or using a cluster as a unit:

(1) Group using ultrasonography in combination: (mammography + ultrasonography, or mammography + clinical breast examination (CBE) + ultrasonography)

(2) Group without ultrasonography: (mammography alone, or mammography + CBE)

2.1 Objectives

To evaluate the diagnostic accuracy and efficacy of breast cancer screening using ultrasonography.

2.2 Study design

The following two groups will be set to evaluate the efficacy of breast cancer screening using ultrasonography in women aged 40 to 49 years:

1) Group using ultrasonography in combination (intervention group)

(mammography + ultrasonography, or mammography + CBE + ultrasonography)

2) Group without ultrasonography (non-intervention group)

(mammography alone, or mammography + CBE).

For scientific evidence, designs such as an individual randomized control study, cluster randomized control study, and non-randomized control study can be assumed. From the viewpoint of performing a high-quality study, an individual randomized control study is the most appropriate followed by a cluster randomized control study. If random allocation cannot be done, a non-randomized control study has to be chosen, and a method to compare with the non-intervention group without randomization has to be considered. A total of 42 organizations in 23 prefectures in Japan will participate in the study.

2.3 Criteria for Selection of Subjects

2.3.1 Age

Women aged 40 to 49 years at the time of enrollment.

2.3.2 Inclusion criteria

In principle, persons who undergo breast cancer screening performed at the study sites will be recruited at the time of screening and requested to provide their informed consent for participation in the study. No special criterion will be specified for a history of breast cancer screening. However, if subjects underwent the screening, the most recent year when they underwent the screening and screening methods should be recorded.

2.3.3 Exclusion criteria

(1) Persons who have a history of breast cancer (including cancer in situ).
(2) Persons who have a history of malignancies except for breast cancer within 5 years (including cancer in situ).
(3) Persons with serious systemic disease who are not expected to survive for at least the next 5 years.

2.4 Endpoints

2.4.1 Primary endpoints

Differences in sensitivity/ specificity and a detection rate of the first breast cancer screening between the intervention group and non-intervention group.

2.4.2 Secondary endpoint

Difference in cumulative incidence of advanced breast cancer during the follow-up period in between the intervention and non-intervention groups.

2.5 Sample size

<u>Primary endpoints:</u>

The primary endpoints are defined as differences in sensitivity/ specificity and a detection rate of the first breast cancer screening between the intervention group and non-intervention group as previously mentioned. The sample size was calculated based on a previous study comparing sensitivity between screening with CBE alone and mammography in combination with CBE.

In this study that was used for understanding interval cancer (cancer identified in screened individuals without abnormality during a period between the screening and the next screening) in population-based cancer registry data in Miyagi Prefecture, the sensitivity of breast cancer screening using mammography in combination with CBE was superior to that of CBE alone in all age groups. However, the sensitivity was 71.4% for the age of 40 to 49 years, 85.8% for the age of 50 to 59 years, and 87.2% for the age of 60 to 69 years; thus, it was lowest for the 40’s. Of 20,587 subjects aged 40 to 49 years, breast cancer occurred in 63 subjects during two years after screening including screen-detected breast cancer. Of these, screen-detected breast cancer was in 45 subjects, and interval cancer was in 18 subjects (sensitivity: 45/63 = 71.4%).

Furthermore, assuming that a combination with ultrasonography would increase the number of subjects with screen-detected cancer and that the incidence of interval cancer would be decreased by half (decrease from 18 to 9

subjects), sensitivity in the ultrasonography combined group would be 86% (54/63). A total of 130 subjects with breast cancer would be necessary for demonstrating this increase in sensitivity with a two-sided significance level of 5% at a power of 80%. When it was converted to the number of subjects undergoing screening, a sample size of 42,500 subjects per group was estimated.

Secondary endpoint:

The secondary endpoint is defined as difference in the cumulative incidence rate of advanced breast cancer during the follow-up period between the intervention and non-intervention groups as previously mentioned. As with the primary endpoints, the sample size was calculated based on the previous study comparing sensitivity between screening with CBE alone and mammography in combination with CBE.

In the above-mentioned mammography group, 12 subjects (27.7%) and 6 subjects (30.6%) had screen-detected breast cancer and advanced breast cancer as interval cancer respectively, and the cumulative incidence of advanced breast cancer was 87 per 100000 (18/20587). For this ratio of advanced breast cancer, assuming that the number of subjects with advanced breast cancer as interval cancer would be also decreased by half (decrease from 6 to 3 subjects) in the ultrasonography combined group, the cumulative incidence of advanced breast cancer in the ultrasonography combined group would be 74 per 100000 (15/20587). A total of 760000 subjects per group would be necessary for demonstrating this with a two-sided significance level of 5% at a power of 80%.

In addition, when the survival rates of screen-detected early breast cancer, screen-detected advanced breast cancer, early interval breast cancer, and advanced interval breast cancer would be 96%, 85%, 92% and 50%, respectively, the cumulative mortality of breast cancer in the mammography group and ultrasonography combined group would be 34 per 100000 and 26 per 100000, respectively. A total of 760000 subjects per group would be necessary for demonstrating these with a two-sided significance level of 5% at a power of 80%. Therefore, a study using the cumulative incidence of advanced breast cancer or the mortality of breast cancer with provision of one screening session as the endpoints is substantially meaningless in terms of statistics.

In contrast, of interval cancers, if the ratio of advanced cancer could be decreased up to 10%, the cumulative mortality of breast cancer in the mammography group and ultrasonography combined group would be 34 per 100000 and 22 per 100000, respectively (reduction in the mortality by 36%). Thus, the final outcome of this study shown by Kurokawa Group, “a decrease in the mortality of breast cancer by 30%” could be achieved. If screening would be continued once every two years for 10 years, the cumulative mortality of breast cancer in each group would be 170 per 100000 and 110 per 100000, respectively. A total of 55000 screened subjects per group would be necessary for demonstrating these with a two-sided significance level of 5% at a power of 80%.

3. Planned Period of the Study

3.1 Schedule of the first and second screening and the number of subjects to be enrolled

	2007	2008	2009	2010	2011	2012
Intervention group	A1 (5000 subjects)	A2 (15000 subjects)	A1 (5000 subjects)	A2 (15000 subjects)		
			A3 (15000 subjects)	A4 (5000 subjects)	A3 (15000 subjects)	A4 (5000 subjects)
Non-intervention group	B1 (5000 subjects)	B2 (15000 subjects)	B1 (5000 subjects)	B2 (15000 subjects)		
			B3 (15000 subjects)	B4 (5000 subjects)	B3 (15000 subjects)	B4 (5000 subjects)
Cumulative No. of subjects newly enrolled	10000 subjects	40000 subjects	70000 subjects	80000 subjects		
Cumulative No. of subjects undergone the second screening			10000 subjects	40000 subjects	70000 subjects	80000 subjects
No. of subjects screened each year	10000 subjects	30000 subjects	40000 subjects	40000 subjects	30000 subjects	10000 subjects

A1, B1: enrolled in 2007, and re-screened in 2009.

A2, B2: enrolled in 2008, and re-screened in 2010.

A3, B3: enrolled in 2009, and re-screened in 2011.

A4, B4: enrolled in 2010, and re-screened in 2012.

3.2 Secondary endpoint

Base on interim analyses which were made in March 2018, data for evaluation will be collected until March 2022. If study expenses can be obtained, the study period may be extended based on the plan.

4. Data Monitoring

In this study, with the use of the central data monitoring system, it will be verified whether the study is conducted safely and in accordance with the protocol, as well as data are correctly collected. After the start of study enrollment, the central data center will, in each month, tabulate the input status of data sent from the Electronic Data Capturing (EDC) shuttle at each site for the quality control (QC) of the data. The central data center will perform periodic logical checks and request the study sites to report cross-checking with source documents with respect to error data. Entry and transcription mistakes identified through cross-checking with source documents will

be corrected by the persons in charge at the study sites. In addition, “data monitoring reports” based on the data collection/ accumulation status and the results of data monitoring will be prepared twice a year and notified to the data monitoring committee. The quality assurance (QA) of the data will be implemented by on-site audits.

5. Definitions and Data Handling Rules for Analyses

5.1 Definitions of screening intervals, breast cancer detected at the first screening, and interval breast cancer

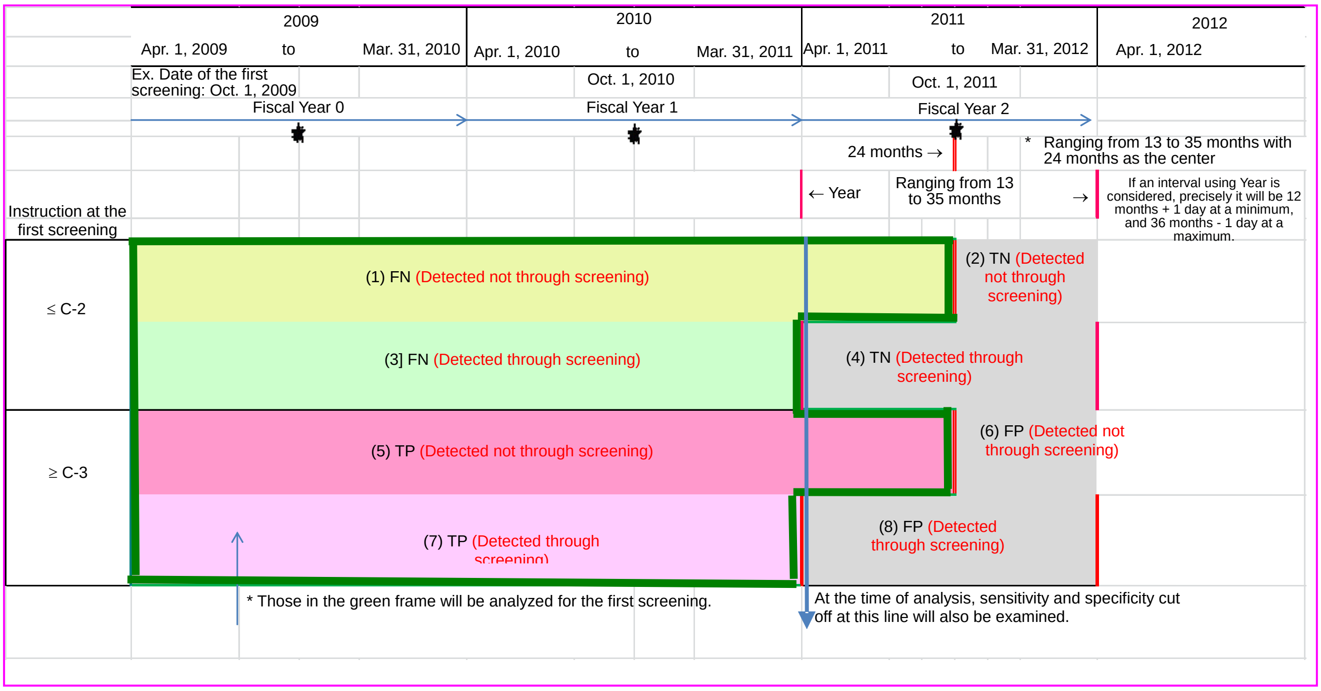

Figure 1. Definitions of screening intervals, breast cancer detected at the first screening, and interval breast cancer

Consensus defined as below was reached at a statistical analysis/ data monitoring joint committee meeting held on July 31, 2013 as a results of several discussions based on breast cancer screening guidelines in Japan as well as the EU and US:

(1) False negative is defined as subjects with ≤ Category 2 assessed at the first screening and with cancer detected not through screening (e.g. medical examination due to subjective symptoms) before 24 months from the date of the first screening.

(2) True negative is defined as subjects with ≤ Category 2 assessed at the first screening and with cancer detected not through screening (e.g. medical examination due to subjective symptoms) after 24 months from the date of the first screening.

(3) False negative is defined as subjects with ≤ Category 2 assessed at the first screening and with cancer detected through screening (including the first screening) before the planned screening in Fiscal Year 2.

(4) True negative is defined as subjects with ≤ Category 2 assessed at the first screening and with cancer detected through screening after the planned screening in Fiscal Year 2.

(5) True positive is defined as subjects with ≥ Category 3 assessed at the first screening and with cancer detected not through screening (e.g. medical examination due to subjective symptoms) before 24 months from the date of the first screening.

(6) False positive is defined as subjects with ≥ Category 3 assessed at the first screening and with cancer detected not through screening (e.g. medical examination due to subjective symptoms) after 24 months from the date of the first screening.

(7) True positive is defined as subjects with ≥ Category 3 assessed at the first screening and with cancer detected through screening (including the first screening) before the planned screening in Fiscal Year 2.

(8) False positive is defined as subjects with ≥ Category 3 assessed at the first screening and with cancer detected through screening after the planned screening in Fiscal Year 2.

5.2 Rules for determining the presence or absence of breast cancer

Breast cancers were ascertained by diagnostic assessment of first and second screening results, hospital discharge records and cancer registry databases. Miyagi prefecture has a sophisticated system of local registration for cancer, which contains virtually all breast cancer patients' registration conducted in Miyagi prefecture. Therefore, it was possible to identify screening-detected cancer, interval cancers and not breast cancer exactly. The latest date for the censoring of data on breast cancer for the purpose of analysis will till February 2020.

5.2.1 Had Breast Cancer (cancer detected at the first screening, interval cancer)

Based on the above-mentioned definitions of screening intervals, cancer detected at the first screening, and interval cancer, subjects classified into (1) FN, (3) FN, (5) TP or (7) TP in the green frame will be used for calculating sensitivity of the first screening.

5.2.2 Did not have Breast Cancer (not breast cancer)

Subjects without breast cancer will be defined as those who do not meet the abovementioned Had Breast Cancer and meet any of the following criteria:

a. Data at the second screening are inputted, and no breast cancer is reported.
b. Questionnaire data are inputted, and no breast cancer is reported.
c. Data at the second screening are missing (screening and questionnaire), but during an interval between the first to second screening, at least one detailed examination is performed, and the results of the detailed examination revealed no breast cancer.
d. Subjects with a diagnosis of breast cancer after the screening interval specified in this study: Subjects who meet (2) TN, (4) TN, (6) FP or (8) FP outside the green frame.

5.2.3 Had unknown status (unknown)

Subjects who do not fall into either of the above Items 1) Had Breast Cancer or 2) Did not have Breast Cancer will be defined as Had unknown status. Subjects whose medical examination data in a report of breast cancer are not grasped will be classified into unknown.

5.3 Data review in a blinded manner (Case ascertainment and validation)

Subjects with a diagnosis of breast cancer in this study will be defined as those with breast cancer recorded in the column for “Diagnosis” in the report of detailed examination results at the examined medical institution and images and pathological data supporting the diagnosis of breast cancer can be understood. After the input function of the EDC installed at each participating site is locked, the central data center should request each study site to submit copies of source documents on subjects with a diagnosis of breast cancer. In addition, the EDC screen capture of the database and documents, in which the randomized groups are blinded, for review should be also sent to the Study Group Secretariat.

It is a given fact that the classifications ((1) FN to (8) FP) of first screen-detected cancer and interval cancer in subjects with a diagnosis of breast cancer based on the statistical definitions are accurate and valid in reliable analyses. In order to secure the accuracy of classifications, data on subjects with a diagnosis of breast cancer will be reviewed by oncological surgeons (Dr. Akihiko Suzuki and Dr. Masaaki Kawai) in a manner that the randomized groups are blinded.

Essential parameters for carrying out a logical check for the classifications ((1) FN to (8) FP) with SAS using the EDC database will be as follows: Assessment of comprehensive category at the first screening, date of screening (all entered volumes), date of detailed examination (all entered volumes), the results of the detailed examination (all entered volumes), information on the study site performing the detailed examination, the date of diagnosis, and the cause of the diagnosis of breast cancer reported in the questionnaire form (screening, medical examination due to subjective symptoms, and detailed examination [or follow-up] as part of screening performed during the screening period in the study).

Table 1. Blinded review and data check in subjects with a diagnosis of breast cancer for classifying them into detected and interval cancers (source documents and EDC data)

Step 1	Use SAS to perform a logical check of essential parameters in EDC data.
Step 2	Blind the randomized groups recorded in case result reports. Review the case by checking screening, detailed examination and the chronology of medical examination/ logics of the course.<br>For 1) subjects in whom breast cancer determined once is denied based on the results of a medical examination at a cancer base hospital, and 2) subjects who are found to be not having breast cancer because of mistakes with transcription in the CRF and input in the column for diagnosis, clean the data based on the facts, and specify the absence of breast cancer. Retain not only the SAS program but also correspondence tables and source documents.<br>* With regard to missing data on TNM Classification and histological classification, make inquiries again to the participating site and medical institution.
Step 3	Cross-check the results of a review assessment and SAS program calculations.
Step 4	If the results in Step 3 are inconsistent, search for the reasons, and handle the matter through processes from an examination, discussion to agreement.
Step 5	Determine the classification to be used for the analysis through the abovementioned examination processes.
Comments	For classification between screen-detected cancer and interval cancer, both screening data at the study site and data on a history of screening recorded in the questionnaire from by the study participants will be used so that the screening and questionnaire data have equal values.

	However, for some subjects with a complicated data structure, for example, 1) when there are both biennial screening specified in the study and annual screening, 2) the results of several follow-ups are entered as detailed examination data, or 3) subjects who report that they have undergone several screening sessions and/or medical examinations not specified in the study in the questionnaire form, accuracy cannot be sufficiently secured only by classification based on data logical checks using analysis software, SAS. Hence, overall verification activities such as cross-checking between source documents and inputted EDC data will be essential so that the source documents of subjects with a diagnosis of breast cancer should be reviewed.

5.4 TNM stages and histological classification data check

In this study, the TNM stage and histological classification are based on UICC-7 and ICD-10, respectively. In this study, the pathological results of specimens resected after surgery will be used as histological classification data. A total of 3 subjects who refused surgery (n = 1) and had inadequate medical examination data were deemed as missing.

5.5 Breast density classification

In this study, we used cases enrolled from the screening center in Miyagi prefectures, as we could examine and confirm the breast density. Mammography density measure was evaluated depending on the fifth edition of Breast Imaging Reporting and Data System (BI-RADS), visual judgment data were classified as follows: (a) almost entirely fatty; (b) scattered areas of fibro-glandular density; (c) heterogeneously dense, which may obscure detection of small masses; and (d) extremely dense, which lowers the sensitivity of mammography screening.

Three expert physicians revaluated mammographic density of the first screening. No personal identifiers were contained in the digital mammograms. All information of screening results, medical check and follow up data were blinded.

6. Analysis Methods

6.1 Statistical analyses of the primary endpoints

This plan describes the analysis of the primary endpoints. Not only to compare between intervention and control group but from a different angle, focusing on breast density and ability of each modality.

The objectives of this study include;

1# To estimate and compare the sensitivity, specificity, screen-detected cancers and interval cancers of the study group and control group.

2# To compare the sensitivity, specificity, screen-detected cancers and interval cancers between dense and non-dense group.

3# To estimate sensitivity of each modality according to breast densities and study groups.

4# To estimate clinical stage and histological findings of screen-detected cancers and interval caners according to breast density.

5# To estimate recall rate and biopsy rate of each modality according to study group.

6.2 Pooling data for analyses

First, the appropriateness of a pooled analysis in subjects who are randomized individually or by cluster will be examined.

In this analysis, study participants assigned by individual randomization and cluster randomization will be analyzed. Cluster randomization in the study will be as follows: Communities or healthcare providers are a randomization unit; explanation and consent are given individually; interventional effect is on an individual basis; and subjects with a diagnosis of breast cancer are those with objective data from a medical examination.

For the estimation of the primary endpoints, generalized estimating equation (GEE) with a compound symmetry structure as a working correlation matrix will be used. Analyses including only subjects who are randomized individually or by cluster will be performed, and it will be reviewed that the point estimates of the primary endpoints are similar. Furthermore, a model including the randomized groups as well as an interaction between the randomization methods (either individual or cluster) and randomized groups as an explanatory variable will be applied to subjects pooled from the two analyses, and it will be evaluated that the interaction effect is not statistically significant. If there is no interaction, a pooled analysis including subjects who are randomized individually and by cluster will be carried out.

6.3 Analysis set

Efficacy analyses will be performed in the intention-to-treat (ITT) set. The ITT set will be a set of participants enrolled in the study excluding (1) non-randomized enrolled subjects, (2) ineligible subjects, (3) subjects withdrawn consent, (4) double-entry subjects, and (5) subjects without data that should be analyzed for demonstrating efficacy (subjects without first screening data).

After all input, correction and sending functions of EDC for study data at the study site are locked, logical check and inquiries to applicable study sites will be made based on data received by the EDC system at the central data center. The final review of the concerned subjects will be carried out based on confirmation, responses and reports from the study sites and source documents.

6.4 Preparation of SAS data set for analysis (Data Source)

When preparing an analysis data set, the below-mentioned verification should be conducted.

6.4.1 Verification of compliance with randomization

After the EDC input function at the site is locked, the key will be opened. System numbers issued by the central randomization and the randomized groups, and system numbers inputted in the EDC at the site and the randomized groups will be cross-checked using data received by the EDC system at the central data center, and inquiries on the following items should be made to each study site:

(1) Verification of subjects incompliant with randomization who underwent screening different from that centrally randomized.

(2) Verification of a system number that a number issued by central randomization is not entered in the EDC.

(3) Verification of subjects for whom numbers different from those assigned by the central data center are inputted in the EDC.

(4) Verification of a system number that has been entered once but deleted.

(5) Double entry

(6) Subjects whose date of consent for the study participation is after the date of screening.

6.4.2 Subjects not meeting the eligible criteria

(1) History of breast cancer

When it is confirmed that there is no difference between EDC input data and source documents entered by the participants, the subjects should be excluded from the analysis.

(2) Other cancers within 5 years

When it is confirmed that there is no difference between EDC input data and source documents entered by the participants, the subjects should be excluded from the analysis.

(3) Age

This study was conducted mainly on the basis of resident screening and occupational screening by local municipalities. For the standard age qualifying breast cancer screening in Japan, age reached in the Year or age at last birthday was used so that the age of 40 ± 2 - 50 ± 2 years would be analyzed.

6.4.3 Withdrawal of consent

Subjects who completely withdraw consent (when cross-checking with external data is refused) will be excluded from the analysis.

6.4.4 Deviation

(1) Subjects without first screening data

For subjects whose participation/ enrollment is recorded but first screening data are not inputted, in accordance with the Section <5.2.1 Full Analysis Set> of the ICH Guideline “Statistical Principles for Clinical Trials,” subjects without data at the time of intervention (i.e., without first screening data) should be deemed as meeting exclusion from the ITT analysis as well thus will be excluded from the analyses of sensitivity and specificity of the first screening.

(2) Subjects with inadequate test (deviation)

i. Subjects in the intervention group who have no ultrasonographic data.

ii. Subjects in the non-intervention group whose ultrasonographic data are inputted.

7. Statistical Analyses

7.1 Comparison of characteristics of baseline demographic variables and he distributions of baseline factors

Baseline demographic variables will be age at the time of enrollment, history of breast cancer screening (presence or absence, date of screening and methods), the age of first menstruation, menstruation status, number of pregnancies, frequency of childbearing, age at the first delivery, feeding history, family history of breast cancer, history of breast surgery, and history of mammary gland disease.

Age at the time of enrollment and time (month) from the previous screening. History of screening, menstruation status, feeding history, family history, history of breast surgery, and history of mammary gland disease. Time (month) from the previous screening, which is a continuous variable, will be categorized. The age of first menstruation, number of pregnancies, frequency of childbearing, age at the first delivery, and number of first-degree relatives with breast cancer will be categorized in accordance with the Gail model for performing an international comparison.

For characteristics of baseline demographic variables, descriptive statistics will be calculated to describe the data. Continuous variables will be summarized with a number, mean, standard deviation, range and mode. Categorical data will be presented as percentages. For methods of comparisons of the distributions of baseline factors, differences in categorical data will be assessed by Fisher's exact test and the Chi-square test, and differences in continuous data by the Mann-Whitney's U-test. All tests will be two-sided.

7.2 Analyses of a detection rate, sensitivity and specificity of the first screening and calculation methods for 95% confidence intervals (CIs)

7.2.1 Definitions and calculation formulas

Table 2. Definitions and calculation formulas of a detection rate, sensitivity and specificity of the first screening

Detection rate of screening, ‰ (95% CI)	The number of cancers with a positive initial interpretation among 1,000 screening examinations.	
Sensitivity, % (95% CI)	$\text{Sensitivity} = \frac{[5]TP+[7]TP}{[5]TP+[7]TP+[1]FN+[3]FN}$	(Number of true positive assessments) / (Number of all positive assessments)
Specificity, % (95% CI)	$\text{Specificity} = \frac{TN}{TN+FP}$	(Number of true negative assessments) / (Number of all negative assessments)

The 95% CIs will be calculated using the binomial distribution method.

At the statistical analysis/ data monitoring committee meeting held on October 28, 2013, consensus was reached that subjects having an unknown status would be excluded from the analyses of sensitivity and specificity.

7.2.2 Method of the primary analysis taking account of correlations within the cluster

All "screen-detected cancer," "interval cancer" and "subjects without cancer" at the first screening to be used for calculating sensitivity/ specificity, the primary endpoints, are binary outcomes. As an analysis method for estimating the primary endpoints in the two groups, GEE taking account of correlations within the cluster will be used. For estimation of the parameters, robust variance estimator with a compound symmetry structure as a working correlation matrix will be employed.

8. Statistical Analysis Rules

Item		Display digit
Subject background characteristics, sensitivity, specificity, detection rate	No. of subjects	To be displayed as integers.
	Proportion (percentage)	To be rounded to the nearest tenth and displayed up to one digit.
	95% CI for the rate (percentage)	
	Detection	To be displayed as ‰
Descriptive statistics	No. of subjects	To be displayed as integers.
	Mean, standard deviation, median	Significant digits will be rounded to the nearest tenth and displayed up to one digit.
	Minimum	The same number of figures as significant digits will be displayed.
	Maximum	The same number of figures as significant digits will be displayed.
	Interquartile range	The same number of figures as significant digits will be displayed.
Test/ estimation	P value	Rules specified by a journal, to which results will be submitted, will be followed. The value will be rounded off to the nearest thousandth and displayed up to three decimal places. However, when it is less than 0.001, it will be displayed as < 0.001.
	Significance level	A two-sided test will be performed with a significance level of 5%. If other values are used for significance levels, they will be mentioned at each time. The confidence coefficient of interval estimation will be two-side 95%.
	CI	Significant digits will be rounded to the nearest tenth and displayed up to one digit.

9. Technical Matters

For all analyses, SAS® software, Version 9.4 (SAS Institute Inc., Cary, NC) will be used.

10. Validation of Analysis Results

For the statistical analyses in this study, under the supervision of both statistical analysis/ data monitoring committees, data sets and SAS programs to be applied to the statistical analyses will be prepared, and analysis operations will be carried out. If data handling is not specified in the analysis plan or other relevant documents, a request for decision and consultation should be, as necessary, made to the statistical analysis/ data monitoring committees, and data should be processed upon consensus.

The validity of the analysis SAS program should be ensured by review, preparation, execution, validation and verification of an SAS program for each analysis using double programming. Practical analysis operations will be performed by two staffs. One has experience with SAS for 14 years (Tohoku University, Ying-Fang Zheng M.D., Ph.D., CCRP® (Certification of Clinical Research Professionals by SoCRA), and the other has experience with SAS for 21 years (J-CRSU, Akihide Inoue, SAS Certified with SAS Base Programmer for SAS®9).

In the program verification and validation, the following items should be checked that: (1) data are the latest; (2) the program to be executed is the latest; (3) if multiple programs are involved, the order of the analysis is correct; (4) date of execution; (5) no error or warning is displayed on the SAS log in the course of execution; and (6) numerical values in a report prepared as output results are consistent.

The analysis plan should be implemented upon confirmation with Professor, Shinichi Kuriyama (Tohoku University) and Professor, Takuhiro Yamaguchi (Tohoku University). Specific instructions and consultations on the analysis methods should be requested to Professor, Yasuo Ohashi (the University of Tokyo/ Chuo University/ J-CRSU), Professor, Takuhiro Yamaguchi (Tohoku University), Professor, Tomotaka Sobue (Osaka University), and Seiichiro Yamamoto, MD. (National Cancer Center). For verification of medical and clinical meaning of data classification, directions should be sought from Professor, Takanori Ishida, (Tohoku University) and Professor, Akihiko Suzuki, (Tohoku Medical and Pharmaceutical University).

11. Changes to Planned Analyses

At a statistical analysis/ data monitoring committee meeting held on January 27, 2014, it was decided to exclude non-randomized subjects from the analyses of the primary endpoints.

12. List of the Statistical Analysis Committee Members

◎ Shinichi Kuriyama	Professor, International Research Institute of Disaster Science, Department of Disaster Public Health, Graduate School of Medicine, Tohoku University
Akira Fukao	Executive Director/ Vice President, Department of Public Health, Yamagata University Faculty of Medicine
Daisuke Shibuya	Director, Cancer Detection Center, Miyagi Cancer Society
Yasuo Ohashi	Professor, Department of Biostatistics, School of Public Health, Graduate School of Medicine, the University of Tokyo
Seiichiro Yamamoto	Director, Public Health Policy Research Division, Research Center for Cancer Prevention and Screening, National Cancer Center
Takuhiro Yamaguchi	Professor, Division of Biostatistics, Graduate School of Medicine, Tohoku University
Ying-Fang Zheng	Researcher, Department of Breast and Endocrine Surgical Oncology, Graduate School of Medicine, Tohoku University
Yoko Narikawa-Shiono	Researcher, Department of Breast and Endocrine Surgical Oncology, Graduate School of Medicine, Tohoku University

(◎ Chairperson)

Statistical Analysis Plan Development Working Group

◎ Shinichi Kuriyama	Professor, International Research Institute of Disaster Science, Department of Disaster Public Health, Graduate School of Medicine Tohoku University
Takuhiro Yamaguchi	Professor, Division of Biostatistics, Graduate School of Medicine, Tohoku University
Akihiko Suzuki	Professor, Department of Breast and Endocrine Surgery, Tohoku Medical and Pharmaceutical University
Masaaki Kawai	Senior Head Physician, Department of Breast Oncology, Miyagi Cancer Center Hospital
Ying-Fang Zheng	Researcher, Department of Breast and Endocrine Surgical Oncology, Graduate School of Medicine, Tohoku University
Yoko Narikawa-Shiono	Researcher, Department of Breast and Endocrine Surgical Oncology, Graduate School of Medicine, Tohoku University

(◎ Chairperson)

13. Development Group for Analysis Procedures for First Screening Results/ Operation Records

Akihiko Suzuki	Professor, Department of Breast and Endocrine Surgery, Tohoku Medical and Pharmaceutical University
Masaaki Kawai	Senior Head Physician, Department of Breast Oncology, Miyagi Cancer Center Hospital
Ying-Fang Zheng	Researcher, Department of Breast and Endocrine Surgical Oncology, Graduate School of Medicine, Tohoku University
Yoko Narikawa-Shiono	Researcher, Department of Breast and Endocrine Surgical Oncology, Graduate School of Medicine, Tohoku University

14. Breast Density Classification Quality Manager Team

Akihiko Suzuki	Professor, Department of Breast and Endocrine Surgery, Tohoku Medical and Pharmaceutical University
Takanori Ishida	Professor, Department of Breast and Endocrine Surgical Oncology, Graduate School of Medicine, Tohoku University
Narumi Harada-Shoji	Assistant Professor, Department of Breast and Endocrine Surgical Oncology, Graduate School of Medicine, Tohoku University
Akiko Sato-Tadano	Assistant Professor, Department of Breast and Endocrine Surgical Oncology, Graduate School of Medicine, Tohoku University

15. Summary of changes from version 1.0 to version 3.0

Section	Original Text	Revised Text	Reason for change
Cover page	Version 1	Version 1.1	
	2013/12/31	2014/1/27	
11. Changes to Planned Analyses	At a statistical analysis/ data monitoring committee meeting held on January 27, 2014, it was decided to exclude non-randomized subjects from the analyses of the primary endpoints.		Added details
Cover page	Version 1.1	Version 2.0	
	2014/1/27	2018/8/20	
6.1 Statistical analyses of the primary endpoints	This plan describes the analysis of the primary endpoints. Not only to compare between intervention and control group but from a different angle, focusing on breast density and ability of each modality.<br>The objectives of this study include;<br>1# To estimate and compare the sensitivity, specificity, screen-detected cancers and interval cancers of the study group and control group.<br>2# To compare the sensitivity, specificity, screen-detected cancers and interval cancers between dense and non-dense group.<br>3# To estimate sensitivity of each modality according to breast densities and study groups.<br>4# To estimate clinical stage and histological findings of screen-detected cancers and interval caners according to breast density.<br>5# To estimate recall rate and biopsy rate of each modality according to study group.		Added details analyses of the sub study to make text clearer.

<table>
<tr><td>5.2 Rules for determining the presence or absence of breast cancer</td><td colspan="2">Breast cancers were ascertained by diagnostic assessment of first and second screening results, hospital discharge records and cancer registry databases. Miyagi prefecture has a sophisticated system of local registration for cancer, which contains virtually all breast cancer patients' registration conducted in Miyagi prefecture. Therefore, it was possible to identify screening-detected cancer, interval cancers and not breast cancer exactly. The latest date for the censoring of data on breast cancer for the purpose of analysis was February 2020.</td><td>To make text clearer.</td></tr>
<tr><td>5.5 Breast density classification</td><td colspan="2">In this study, we used cases enrolled from the screening center in Miyagi prefectures, as we could examine and confirm the breast density. Mammography density measure was evaluated depending on the fifth edition of Breast Imaging Reporting and Data System (BI-RADS), visual judgment data were classified as follows: (a) almost entirely fatty; (b) scattered areas of fibro-glandular density; (c) heterogeneously dense, which may obscure detection of small masses; and (d) extremely dense, which lowers the sensitivity of mammography screening.
Three expert physicians revaluated mammographic density of the first screening. No personal identifiers were contained in the digital mammograms. All information of screening results, medical check and follow up data were blinded.</td><td>To make text clearer.</td></tr>
<tr><td rowspan="2">Cover page</td><td>Version 2.0</td><td>Version 3.0</td><td rowspan="2"></td></tr>
<tr><td>2018/8/20</td><td>2019/1/18</td></tr>
<tr><td>14. Breast Density Classification Quality Manager Team</td><td colspan="2">Akihiko Suzuki Professor, Department of Breast and Endocrine Surgery, Tohoku Medical and Pharmaceutical University
Takanori Ishida Professor, Department of Breast and Endocrine Surgical Oncology, Graduate School of Medicine, Tohoku University
Narumi Harada-Shoji Assistant Professor, Department of Breast and Endocrine Surgical Oncology, Graduate School of Medicine, Tohoku University</td><td>Update membership</td></tr>
</table>

	Akiko Sato-Tadano Assistant Professor, Department of Breast and Endocrine Surgical Oncology, Graduate School of Medicine, Tohoku University	
