# Supplementary material for: Evaluation of Adjunctive Ultrasonography for Breast Cancer Detection Among Women Aged 40-49 Years With Varying Breast Density Undergoing Screening Mammography: A Secondary Analysis of a Randomized Clinical Trial
Source: JAMA Netw Open. 2021 Aug 18;4(8):e2121505. doi: 10.1001/jamanetworkopen.2021.21505 (PMC8374606; doi:10.1001/jamanetworkopen.2021.21505)
Supplement: Supplement 2. — eFigure. Trial Profile eTable. Distribution of Mammography Density Based on 5th Edition BI-RADS Density Categories [file jamanetwopen-e2121505-s002.pdf]

Supplemental Online Content

Harada-Shoji N, Suzuki A, Ishida T, et al. Evaluation of adjunctive ultrasonography for breast cancer detection among women aged 40-49 years with varying breast density undergoing screening mammography: a secondary analysis of a randomized clinical trial. *JAMA Netw Open*. 2021;4(8):e2121505. doi:10.1001/jamanetworkopen.2021.21505

eFigure. Trial Profile
eTable. Distribution of Mammography Density Based on 5th Edition BI-RADS Density Categories

This supplemental material has been provided by the authors to give readers additional information about their work.

© 2021 Harada-Shoji N et al. *JAMA Network Open.*

eFigure. Trial Profile

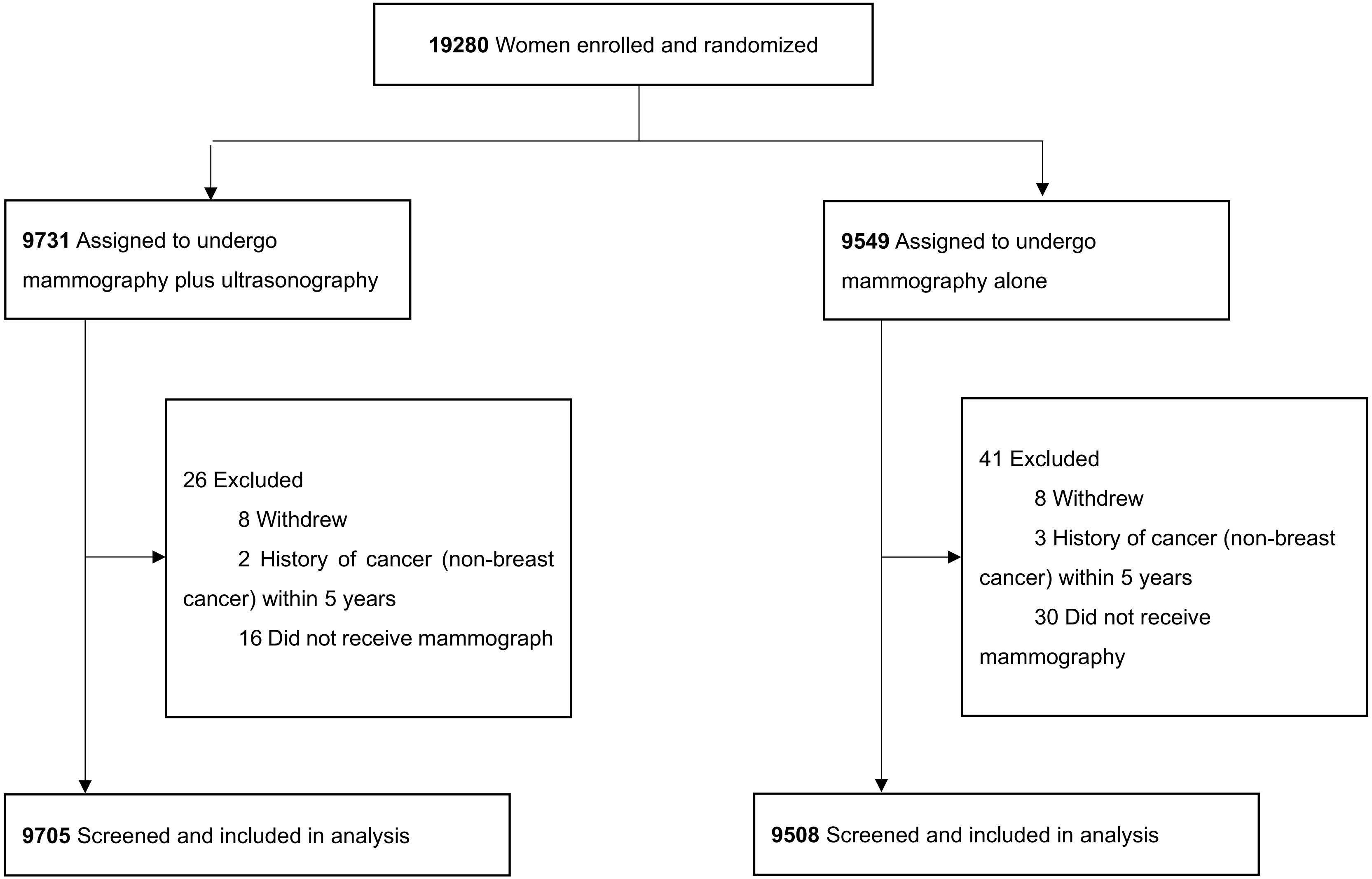

© 2021 Harada-Shoji N et al. *JAMA Network Open.*

eTable. Distribution of Mammography Density Based on 5th Edition BI-RADS Density Categories.

	Total participants (N = 19213)	Intervention group (N = 9705)			Control group (N = 9508)		
		No breast cancer	Screen-detected cancers	Interval cancers	No breast cancer	Screen-detected cancers	Interval cancers
Extremely dense, No. (%)	1371 (7.1)	698 (7.2)	5 (0.05)	0	662 (7.0)	2 (0.02)	4 (0.04)
Heterogeneously dense, No. (%)	10019 (52.2)	5055 (52.1)	36 (0.37)	3 (0.03)	4897 (51.5)	22 (0.23)	6 (0.06)
Scattered fibroglandular tissues, No. (%)	7283 (37.9)	3593 (37.0)	27 (0.28)	2 (0.02)	3640 (38.3)	13 (0.14)	8 (0.08)
Almost entirely fatty, No. (%)	540 (2.8)	286 (3.0)	0	0	252 (2.7)	1 (0.01)	1 (0.01)

Abbreviation: BI-RADS: Breast Imaging Reporting and Data System

© 2021 Harada-Shoji N et al. *JAMA Network Open.*
