# Supplementary material for: Evaluation of Adjunctive Ultrasonography for Breast Cancer Detection Among Women Aged 40-49 Years With Varying Breast Density Undergoing Screening Mammography: A Secondary Analysis of a Randomized Clinical Trial
Source: JAMA Netw Open. 2021 Aug 18;4(8):e2121505. doi: 10.1001/jamanetworkopen.2021.21505 (PMC8374606; doi:10.1001/jamanetworkopen.2021.21505)
Supplement: Supplement 3. — Data Sharing Statement [file jamanetwopen-e2121505-s003.pdf]

Data Sharing Statement

Harada-Shoji. Evaluation of Adjunctive Ultrasonography for Breast Cancer Detection Among Women Aged 40-49 Years With Varying Breast Density Undergoing Screening Mammography. *JAMA Netw Open*. Published August 18, 2021.
doi:10.1001/jamanetworkopen.2021.21505

Data
Data available: Yes
Data types: Deidentified participant data
How to access data: http://www.j-start.org/
When available: With publication

Supporting Documents
Document types: None

Additional Information
Who can access the data: Noriaki Ohuchi, MD, PhD <noriaki-ohuchi@med.tohoku.ac.jp>
Types of analyses: for any purpose
Mechanisms of data availability: with investigator support
